# Impaired Membrane Energetics Underlies Collateral Sensitivity to Amphenicols During Meropenem Resistance Evolution in *Escherichia coli*

**DOI:** 10.3390/microorganisms14081792

**Published:** 2026-08-14

**Authors:** Yinshu Li, Qi Jiang, Tao Zheng, Ruanyang Sun, Hongyan Zhang, Hao Ren, Liangxing Fang, Yufeng Zhou, Jian Sun, Xiaoping Liao

**Affiliations:** State Key Laboratory for Animal Disease Control and Prevention, College of Veterinary Medicine, South China Agricultural University, Guangzhou 510642, China; liyinshu@stu.scau.edu.cn (Y.L.); jiangqi845@163.com (Q.J.); 18948661259@163.com (T.Z.); sunruanyang@163.com (R.S.); qiyue1031@outlook.com (H.Z.); hao.ren@scau.edu.cn (H.R.); fanglx@scau.edu.cn (L.F.); zyf@scau.edu.cn (Y.Z.); jiansun@scau.edu.cn (J.S.)

**Keywords:** collateral sensitivity, resistance evolution, meropenem, amphenicols, *Escherichia coli*

## Abstract

Collateral sensitivity, an evolutionary trade-off accompanying antibiotic resistance, offers a potential strategy to counteract multidrug resistance. Here, we characterized collateral sensitivity profiles in plasmid-bearing and plasmid-free *Escherichia coli* (*E. coli*) lineages evolved under diverse antibiotic selection pressures. Across six independent meropenem (MEM)-evolved lineages derived from two distinct genetic backgrounds, a conserved collateral sensitivity to amphenicols consistently emerged during adaptive laboratory evolution (ALE). Whole genome sequencing identified recurrent mutations in *envZ*, *mrdA* and *yghB*, with *yghB* as an important genetic determinant associated with amphenicol sensitivity. Functional characterization of *yghB* disruption revealed that loss of YghB function affected membrane integrity and proton motive force (PMF) homeostasis, accompanied by altered efflux-associated activity, increased intracellular accumulation of amphenicols and altered Mg^2+^ homeostasis. In the Δ*yghB* background, these alterations were further associated with disrupted ribosome homeostasis and reduced translational capacity. Restoration of Mg^2+^ partially recovered ribosome-associated processes and amphenicol resistance in the Δ*yghB* mutant, suggesting that Mg^2+^ limitation contributes to *yghB*-associated amphenicol sensitivity. Furthermore, amphenicol treatment improved infection outcomes against MEM-resistant mutants in both *Galleria mellonella* and murine septicemia models. Collectively, MEM resistance evolution in *E. coli* generates robust collateral sensitivity to amphenicols, in which recurrent *yghB* mutations represent an important genetic basis. Functional analysis of *yghB* disruption further suggests that altered membrane energetics, efflux-associated processes and Mg^2+^-dependent ribosome homeostasis may contribute to this collateral sensitivity, revealing a potential therapeutic opportunity based on evolutionary trade-offs.

## 1. Introduction

As resistance continues to evolve faster than drug discovery, novel evolution-informed strategies are urgently needed to restore antibiotic efficacy and extend the lifespan of existing agents [1,2]. Antibiotic resistance evolution often imposes adaptive costs, as resistance-associated mutations may compromise bacterial fitness through reduced growth or impaired competitive ability [3,4]. Among these resistance-associated trade-offs, changes in antibiotic susceptibility represent a particularly exploitable form of adaptation, termed collateral sensitivity, in which resistance to one antibiotic results in increased susceptibility to another [5,6]. Recent reviews have highlighted collateral sensitivity as a promising strategy to combat antimicrobial resistance, while emphasizing the importance of understanding its underlying mechanisms, evolutionary robustness, and therapeutic potential [7,8]. Exploiting such predictable collateral sensitivity relationships provides a potential strategy to guide antibiotic cycling or alternating therapy and constrain the evolution of multidrug resistance [6,9]. Although several studies have exhibited collateral sensitivity among aminoglycosides, β-lactams, and tetracyclines [10,11,12], most investigations have been performed in laboratory-adapted strains with relatively simple genetic backgrounds [10,13]. In contrast, clinically relevant strains frequently harbor conjugative resistance plasmids and acquire additional adaptive mutations, which may contribute to altered antibiotic susceptibility profiles and evolutionary trajectories [11,14,15,16]. Among these, extended-spectrum β-lactamase (ESBL)-encoding plasmids, particularly those carrying CTX-M enzymes, have become globally prevalent in *Enterobacteriaceae* and constitute an important source of genetic diversification [17]. Given their contribution to bacterial genetic diversity, whether collateral sensitivity relationships remain conserved across divergent genetic backgrounds, particularly following acquisition of clinically relevant ESBL plasmids, remains largely unexplored.

Despite increasing interest in collateral sensitivity as an evolution-informed therapeutic strategy, recent reviews have highlighted that the robustness and therapeutic potential of collateral sensitivity are shaped by genetic background, resistance mechanisms, and physiological constraints [18,19]. However, the physiological mechanisms governing its emergence and conservation remain incompletely understood, particularly in clinically relevant bacterial backgrounds. Recent evidence suggests that perturbations in membrane homeostasis, proton motive force (PMF), ion transport, and efflux activity can substantially influence bacterial susceptibility to multiple antibiotic classes [13,20]. Because these physiological processes are frequently altered during adaptive evolution, they may represent important but poorly understood determinants of collateral sensitivity. However, how such physiological changes contribute to the establishment and maintenance of collateral sensitivity phenotypes remains largely unclear, particularly in clinically relevant *Escherichia coli* (*E. coli*) backgrounds.

To address these questions, we systematically characterized the collateral sensitivity profiles of plasmid-bearing and plasmid-free *E. coli* lineages generated through adaptive laboratory evolution (ALE) under diverse antibiotic selection pressure. We identified a conserved and robust collateral sensitivity to amphenicols that consistently emerged during meropenem (MEM)-selected ALE. Through genomic, transcriptomic, and functional analyses, we further elucidated the molecular basis of this phenotype and identified mutations in the DedA family membrane protein YghB as a key contributor to amphenicol sensitivity. Mechanistically, disruption of YghB impairs membrane integrity and perturbs PMF homeostasis, leading to reduced efflux activity, altered Mg^2+^ homeostasis, and impaired ribosome homeostasis. Finally, we demonstrated that this conserved collateral sensitivity can be therapeutically exploited in both *Galleria mellonella* and murine infection models. Together, these findings provide mechanistic insight into the evolution of collateral sensitivity and highlight the potential of exploiting robust evolutionary trade-offs for antimicrobial therapy.

## 2. Materials and Methods

### 2.1. Bacterial Strains and Antibiotics

A clinical IncI1-type plasmid p15E179, carrying a chloramphenicol (CHL) resistance gene *catA1* and a cephalosporin resistance gene *bla*_CTX-M-55_, was transferred into *E. coli* C600 by conjugation to generate the plasmid-carrying parental strain (WT; Table S1). The WT-derived MEM-resistant evolved population exhibiting the strongest and most reproducible collateral sensitivity phenotype was designated MEM-R and used for subsequent genomic, transcriptomic, and mechanistic analyses. All antibiotics used in this study, along with their abbreviations, are shown in Table 1.

### 2.2. Laboratory Evolutionary Experiments

Both WT and C600 were used as parental strains for adaptive laboratory evolution (ALE) experiment. Prior to experimental evolution, minimum inhibitory concentration (MIC) of all strains was determined for six antibiotics (MEM, CIP, FOS, DOX, APR and COL). ALE experiment was performed using these six antibiotics as the selection pressure. A single colony of each strain was used to inoculate 500 μL of LB and incubated at 37 °C with shaking at 220 rpm. Each culture was then diluted 1:100 into one antibiotic-free control population and three independent antibiotic-exposed evolutionary populations containing LB with six antibiotics at five concentrations (1/4 to 4 × MIC).

All populations were incubated for 24 h at 37 °C, followed by daily 1:100 transfers into fresh LB containing antibiotics. Parallel control populations were passaged without antibiotics to account for adaptations unrelated to drug exposure. Each day, the highest antibiotic concentration supporting visible growth (OD_600_ > 0.075) was recorded as the daily MIC. This procedure was repeated for 14 consecutive days. At the end of the evolution experiment, evolved mutants were subcultured for three passages in antibiotic-free LB to eliminate transient tolerance and confirm stable resistance. Final MICs were determined to verify resistance acquisition. Both evolved and control strains were preserved at −80 °C in 30% (*v*/*v*) glycerol for subsequent analysis.

### 2.3. Antimicrobial Susceptibility Testing

To evaluate collateral effects and trade-offs in resistance evolution, antimicrobial susceptibility was determined for the parental strains and their evolved derivatives. Susceptibility was assessed against 14 antibiotics representing major mechanistic classes commonly used in clinical practice (Table 1). MIC was performed using the broth microdilution method according to the CLSI guidelines [21]. Antimicrobial susceptibility categories (susceptible, intermediate, or resistant) were interpreted according to CLSI and EUCAST clinical breakpoints [21,22]. *E. coli* ATCC 25922 served as the quality control strain. All MIC determinations were performed in three independent biological replicates per antibiotic.

### 2.4. Whole Genome Sequencing (WGS) Analysis

Genomic DNA was extracted directly from each evolved population derived from MEM selection pressure, as well as from the parental strains, using a bacterial genomic DNA extraction kit (Tiangen, Beijing, China). DNA purity and quantification were determined using agarose gel electrophoresis and NanoDrop spectrophotometer (Thermo Scientific, Pittsburg, PA, USA). WGS was performed for three independent MEM-selected evolved populations and their parental strains using the HiSeq 2500 system (Illumina, San Diego, CA, USA) with the paired-end 2 × 150 bp sequencing protocol. The raw sequencing reads of the whole genome sequencing datasets generated in this study have been deposited in the NCBI Sequence Read Archive (SRA) under BioProject accession numbers PRJNA1224587 (WT strain) and PRJNA1226379 (MEM-resistant evolved populations). The reference genome of *E. coli* C600 was obtained from NCBI under BioProject accession PRJNA472644.

Raw reads were quality-checked using FastQC v0.11.9, assembled de novo with SPAdes v3.9.0, and annotated using Prokka v1.14.5. Sequence reads were mapped back to the assembled genomes using Bowtie2 to determine coverage and verify chromosomal integrity. The read mapping rates across all WGS libraries ranged from 97.15% to 99.86%. Downstream analyses included genome component prediction, gene function annotation, and comparative genomic analysis. After excluding mutations present in the ancestral strain and antibiotic-free control populations, recurrent mutations across independent MEM-selected evolved populations were identified as candidate adaptive changes associated with resistance evolution.

### 2.5. Construction of Gene Deletion and Overexpression Strains

Chromosomal gene deletions were generated in the WT strain using the CRISPR/Cas9 system with plasmids and primers (Tables S2 and S3) [23]. For *yghB* knockout construction, upstream and downstream homologous arms flanking the *yghB* locus were amplified and cloned into the pSGKP plasmid carrying the corresponding sgRNA, followed by digestion and assembly using the ClonExpress Ultra One Step Cloning Kit (Vazyme, Nanjing, China). The resulting construct was transformed into *E. coli* DH5α (Tsingke, Beijing, China) and subsequently introduced into pCasKP-harboring WT for allelic exchange. *envZ* and *ynfL* mutants were generated using the same procedure. All deletions were verified by PCR and Sanger sequencing.

The chromosomal *yghB* A645del allele was reconstructed in the WT strain using a two-step CRISPR/Cas9-mediated allelic replacement strategy with plasmids and primers (Tables S2 and S3). Briefly, the native *yghB* locus was first replaced with an antibiotic resistance cassette through homologous recombination using the CRISPR/Cas9 system. Subsequently, the resistance cassette was replaced by the evolved *yghB* frameshift allele using a second round of CRISPR/Cas9-mediated counter-selection. The final recombinant strain was verified by PCR and Sanger sequencing.

For gene overexpression, target coding sequences were amplified and cloned into the pBAD expression vector, which was linearized by reverse amplification. Recombinant plasmids were assembled using the ClonExpress Ultra One Step Cloning Kit (Vazyme, Nanjing, China) and first validated in *E. coli* DH5α before being introduced into WT. Overexpression of genes such as *mrdA*, *envZ* and *yghB* was induced with 0.5% (*w*/*v*) arabinose.

### 2.6. Circular Dichroism (CD) Spectroscopy

To assess the structural differences between wild-type and mutant YghB proteins, plasmids harboring *yghB*^WT^ and *yghB*^FS^ were transformed into *E. coli* BL21 (Tsingke, Beijing, China) for protein expression (Tables S2 and S3). Protein expression, extraction, purification, and refolding were performed using a modified inclusion body purification protocol followed by stepwise dialysis as previously described [24].

CD spectra were recorded using a Chirascan^TM^ CD Spectrometer in PBS buffer (Applied Photophysics Ltd., Leatherhead, UK). Measurements were obtained in a 1 mm path-length quartz cuvette, with spectra collected over the 180–260 nm wavelength range at 0.5 s per point. Each spectrum represents the average of three technical scans. Data analysis was performed using CDNN 2.1, and the secondary structure composition was estimated from the deconvoluted spectra.

### 2.7. Measurements of Membrane Functions, PMF, ATP and Efflux Activity

Overnight bacterial cultures were inoculated into fresh LB containing 0.5% (*w*/*v*) arabinose incubated at 37 °C with 220 rpm for 6 h. Cells were harvested by centrifugation, washed twice with PBS, and resuspended to an OD_600_ of 0.5. These suspensions were used for all subsequent assays. Each assay was performed with at least three independent biological replicates.

(i) Plasma membrane integrity and outer membrane permeability. Bacterial suspensions were incubated separately with propidium iodide (PI, 10 μM; MedChemExpress, Monmouth Junction, NJ, USA) and N-phenyl-1-naphthylamine (NPN, 10 μM; MedChemExpress, Monmouth Junction, NJ, USA) at 37 °C for 1 h. After incubation, 200 μL of each mixture was transferred into a 96-well black plate and fluorescence intensity was measured (PI: excitation 535 nm, emission 615 nm; NPN: excitation 350 nm, emission 420 nm).

(ii) PMF. To assess the transmembrane proton gradient (ΔpH) and membrane potential (Δψ), bacterial suspensions were incubated with BCECF-AM (10 μM; MedChemExpress, Monmouth Junction, NJ, USA) or DiSC_3_(5) (10 μM; MedChemExpress, Monmouth Junction, NJ, USA) at 37 °C for 1 h. Fluorescence was recorded in a 96-well black plate (BCECF-AM: excitation 490 nm, emission 535 nm; DiSC_3_(5): excitation 622 nm, emission 670 nm).

(iii) ATP. Intracellular ATP levels in bacterial cultures were quantified using the Enhanced ATP Assay Kit (Beyotime, Shanghai, China) according to the manufacturer’s instructions. Briefly, bacterial cultures were collected and processed for ATP extraction, and luminescence signals were measured to determine ATP concentrations. ATP levels were normalized to bacterial biomass and expressed relative to the WT strain.

(iv) Efflux activity. Bacterial suspensions were incubated with ethidium bromide (EtBr, 5 μM; MedChemExpress, Monmouth Junction, NJ, USA) in the presence or absence of efflux inhibitors, including carbonyl cyanide m-chlorophenylhydrazone (CCCP, 20 μM; MedChemExpress, Monmouth Junction, NJ, USA), Phe-Arg-β-naphthylamide (PAβN, 25 μM; MedChemExpress, Monmouth Junction, NJ, USA), or reserpine (RSP, 200 μM; MedChemExpress, Monmouth Junction, NJ, USA) for 30 min at 37 °C. Fluorescence was then measured (excitation 530 nm, emission 600 nm).

### 2.8. Measurement of Intracellular Antibiotic Accumulation

Overnight cultures were diluted 1:100 in LB and grown for 6 h at 37 °C with 220 rpm. Three independent biological replicates were used for intracellular antibiotic accumulation assays. Cells were harvested by centrifugation (6500 rpm, 10 min), resuspended in fresh LB to an OD_600_ of 0.5, and incubated with CHL or FFC for 1 h at 37 °C. Cells were then washed twice with PBS by centrifugation (6500 rpm, 10 min) to remove extracellular antibiotic. The pellets were lysed by sonication at 40% amplitude for 30 cycles (3 s on, 5 s off). After centrifugation (12,000 rpm, 10 min, 4 °C), supernatants were filtered through 0.22 μm membranes and transferred to vials for LC-MS/MS analysis (Agilent Technologies, Santa Clara, CA, USA). LC-MS/MS peak areas were converted into antibiotic concentrations using the corresponding calibration curves. The calculated intracellular antibiotic amounts were normalized to the initial OD_600_ value of the bacterial suspension to account for differences in bacterial input and were expressed as relative intracellular antibiotic accumulation levels.

### 2.9. RNA-Seq and Transcriptomic Analysis

Overnight cultures were diluted 1:100 in LB and incubated at 37 °C with 220 rpm for 4~6 h. Total RNA was extracted using the OMEGA Total RNA Kit I (Omega, Suzhou, China) following the manufacturer’s instructions, and RNA purity was assessed by the A260/A280 ratio using a NanoDrop spectrophotometer (Thermo Fisher Scientific, Wilmington, DE, USA). RNA libraries were prepared from three independent biological replicates using an Illumina TruSeq RNA sample prep kit v2 (Illumina Inc., San Diego, CA, USA) according to the manufacturer’s protocol [25]. Briefly, mRNA was fragmented into 200–300 bp fragments, and first- and second-strand cDNA were synthesized, purified, end-repaired, A-tailed, and ligated to sequencing adapters. Sequencing reads were processed using FANse2 software (available at http://www.chi-biotech.com/fanse2/intro.html, accessed on 1 May 2025) and mapped to the *E. coli* reference genome (GenBank accession CP031214) to quantify gene expression. The read mapping rates across all RNA-seq libraries ranged from 98.91% to 99.44%. Differentially expressed genes (DEGs) were identified using a false discovery rate (FDR)  <  0.05 and |log_2_FC| > 1. KEGG enrichment analysis of DEGs was performed using KOBAS (available at http://bioinfo.org/kobas/, accessed on 10 June 2025). The raw RNA-sequencing reads generated in this study have been deposited in the NCBI SRA under BioProject accession numbers PRJNA1224518 (WT and Δ*yghB* strains) and PRJNA1226379 (WT and MEM-R strains).

### 2.10. Reverse Transcription Quantitative PCR (RT-qPCR) Assays

RT-qPCR was performed using the Taq Pro Universal SYBR qPCR Master Mix kit (Vazyme Biotech, Nanjing, China). Cycling conditions consisted of the following: 95 °C for 30 s followed by 40 cycles of 10 s at 95 °C and 30 s at 60 °C. All samples were analyzed in triplicate and the *16S rRNA* gene was used as an endogenous control. Fold changes in gene expression were analyzed by the 2^−∆∆CT^ method. The primers used in this study are listed in Table S3.

For Mg^2+^-dependent transcriptional analysis, WT and Δ*yghB* strains were cultured in standard LB medium supplemented with the indicated concentrations of MgSO_4_. Gene expression levels were normalized to the *16S rRNA* gene and calculated as 2^−∆CT^ for each sample. To compare Mg^2+^-responsive transcriptional changes between Δ*yghB* and WT strains under the same extracellular Mg^2+^ concentration, relative expression values were calculated as the ratio 2^−∆CT^_Δ*yghB*_/2^−∆CT^_WT_ and used for graphical presentation.

### 2.11. Polysome Analysis

Overnight cultures of WT and Δ*yghB* were diluted 1:100 in LB and grown at 37 °C with 220 rpm for 4~6 h. Cells were harvested at comparable mid-exponential growth phase (OD_600_ ≈ 0.6), then lysed, and their polysomes were separated in a sucrose gradient as described [26]. Gradients were fractionated into 30 fractions using a Gradient Station IP fractionator (BioComp, Fredericton, NB, Canada), and the first 27 fractions were analyzed. Absorbance at 260 nm was recorded using a NanoDrop 8000 spectrophotometer (Thermo Scientific, Wilmington, DE, USA). The identity of the 260 nm peaks was verified by phenol:chloroform extraction, ethanol precipitation, and analysis by denaturing agarose gel electrophoresis.

### 2.12. Estimation of RNA/Protein Ratio

Overnight cultures were diluted 1:100 in LB and incubated at 37 °C for 6 h. Cells from three independent biological replicates were washed with PBS and lysed by sonication. Crude extracts were used to determine total RNA and protein contents using the orcinol assay and Pierce BCA Protein Assay Kit (Thermo Fisher Scientific, Wilmington, DE, USA), respectively. The RNA/protein ratio was calculated from the measured concentrations.

### 2.13. Mg^2+^ Quantitation

Overnight bacterial cultures were inoculated into standard LB medium containing 0.5% (*w*/*v*) arabinose and incubated at 37 °C with 220 rpm for 6 h. Cells from three independent biological replicates were collected for intracellular Mg^2+^ quantification. Cells were harvested by centrifugation at 6500 rpm for 10 min, washed twice with PBS, and resuspended in Hanks’ balanced salt solution to an OD_600_ of 0.5. Intracellular Mg^2+^ concentrations were measured using the Mag-Fura-2 AM (MKBio, Shanghai, China) according to the manufacturer’s protocol. Fluorescence intensity was detected at an excitation wavelength of 340 nm and 380 nm, and an emission wavelength of 503 nm. For Mg^2+^ supplementation experiments, MgSO_4_ was added to standard LB medium at the indicated final concentrations before bacterial cultivation.

### 2.14. Short-Term Collateral Sensitivity-Based Treatment Experiments

For short-term antibiotic selection experiments, three replicate populations of WT were cultured daily for three days in LB supplemented with both MEM (0.5 mg/L) and an amphenicol (64 mg/L CHL or 0.5 mg/L FFC). For collateral sensitivity-based treatment experiments, three independent replicate populations of MEM-R were diluted 1:100 daily into fresh LB containing either 64 mg/L CHL or 0.5 mg/L FFC alone. Population extinction was assessed by plating the final cultures on LB agar and examining colony formation.

### 2.15. Galleria mellonella (G. mellonella) Infection Model

Larvae of *G. mellonella* were randomly divided into five groups (*n* = 10) and infected with the WT or MEM-R suspensions (10^7^ CFU/mL, 10 μL) injected into the right posterior proleg. One hour post-infection, larvae were treated with either PBS or FFC (20 mg/kg) injected into the left posterior proleg. The FFC dose was selected based on previously reported FFC dosing regimens [27,28] and calculated based on the average larval body weight (approximately 300 mg per larva, corresponding to 6 μg FFC per larva). Survival was monitored daily for 4 days.

### 2.16. Mouse Peritonitis-Septicemia Model

The Institutional Animal Care and Use Committee of the South China Agricultural University approved all animal studies (approval no. 2025C056). A total of 24 female ICR mice (20 g) of 6 weeks old were randomly assigned into four groups (*n* = 6 per group) and maintained in ventilated cages with free access to food and water. After 3 days of acclimatization, mice were infected intraperitoneally with the WT or MEM-R (0.2 mL of 10^8^ CFU/mL). One hour post-infection, mice received 0.1 mL of FFC (20 mg/kg, corresponding to 0.4 mg per mouse based on an average body weight of 20 g) or PBS intraperitoneally. The selected dose was based on previously reported FFC dosing regimens in animal studies [27,28]. After 12 h, all mice were euthanized, and the liver, spleen, and kidney were aseptically collected, homogenized in PBS, and plated for bacterial load determination. Tissues were also processed for histopathological examination. All histological evaluations were performed by an investigator blinded to the treatment group assignments.

### 2.17. Statistical Analysis

Unless otherwise stated, experiments were performed in at least three independent biological replicates conducted on different days using fresh overnight cultures. A biological replicate was defined as an independent culture initiated from a single colony or an independent evolutionary lineage. Technical replicates were averaged prior to statistical analysis and were not treated as independent observations. All statistical analyses were performed using GraphPad Prism 10.0 (GraphPad Software Inc., La Jolla, CA, USA). Between-group comparisons were conducted using unpaired *t*-tests or one-way ANOVA as appropriate. For one-way ANOVA, Tukey’s multiple comparisons test was used when comparisons among all groups were performed, whereas Dunnett’s multiple comparisons test was applied when each experimental group was compared with the WT control. A *p* value < 0.05 was considered statistically significant.

## 3. Results

### 3.1. Conserved Amphenicol Collateral Sensitivity Across Evolved Populations

Following plasmid acquisition, the WT strain exhibited a high level of resistance to CHL (MIC = 256 mg/L) and cephalosporins (CTX and CEF, MIC = 256 mg/L) while remaining susceptible to other antibiotics. Plasmid carriage did not result in an apparent fitness disadvantage, as the WT and plasmid-free *E. coli* C600 showed comparable growth kinetics.

**Figure 1:**
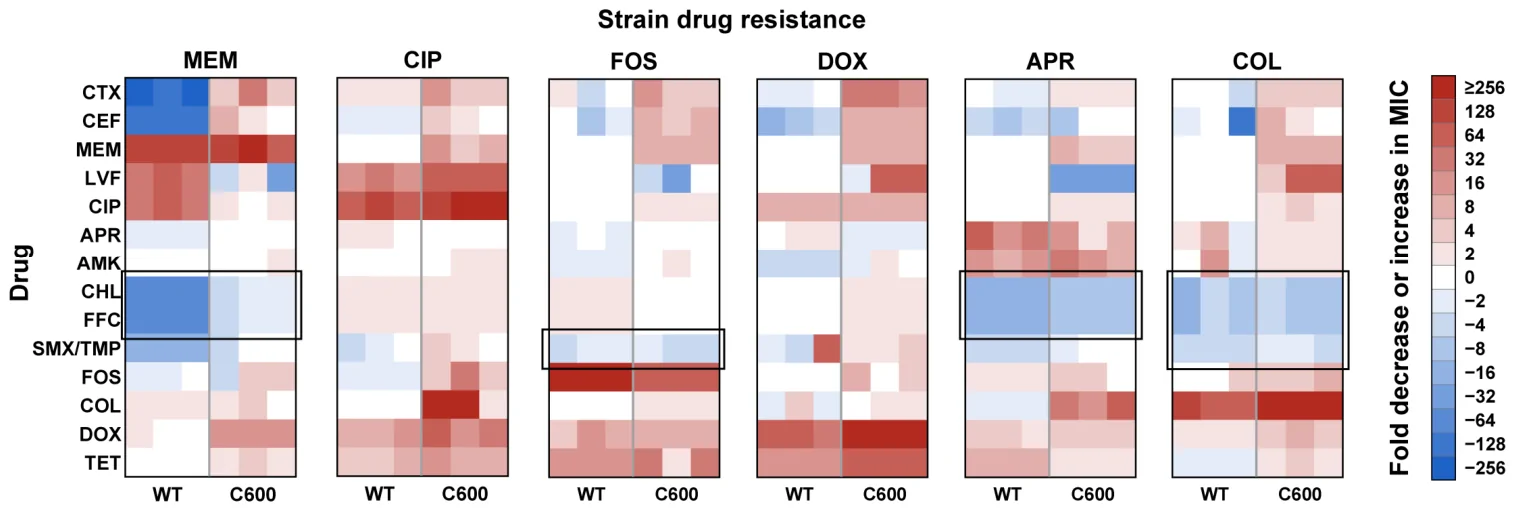
Collateral sensitivity profiling in WT- and C600-derived lineages under antibiotic selection pressure. Heatmap showing the fold change of MIC in evolved mutants relative to each parental strain across multiple clinically relevant antibiotics. The black boxes indicate robust collateral sensitivity phenotypes.

At the end of the ALE experiment, evolved populations from each lineage were collected for antimicrobial susceptibility testing. The profiling revealed distinct collateral sensitivity patterns under different antibiotic selection pressures (Figure 1). Robust and reproducible collateral sensitivity phenotypes were observed among populations evolved under MEM, FOS, APR and COL selection pressures, whereas no consistent patterns were observed under CIP or DOX selection pressure. Notably, increased susceptibility to CHL and FFC emerged as a highly consistent phenotype in both WT- and C600-derived lineages across multiple selection conditions, suggesting a conserved amphenicol-associated collateral sensitivity.

Under MEM selection pressure, WT-derived populations exhibited a 128-fold increase in MEM MIC, accompanied by markedly decreased susceptibility to CHL and FFC (64-fold), as well as to CTX (128~256-fold), CEF (128-fold) and SMX/TMP (16-fold). In comparison, C600-derived MEM-selected populations showed a 64~256-fold increase in MEM MIC and a 2~4-fold decrease toward CHL and FFC. Consistent with this amphenicol-associated collateral sensitivity, similar phenotypes were also observed under APR and COL selection pressure. APR-selected populations displayed 16- and 8-fold reductions in amphenicol MICs in WT- and C600-derived lineages, respectively. COL-selected populations displayed the broadest collateral sensitivity profile, including decreased susceptibility to CHL and FFC (4~16-fold in WT and 4~8-fold in C600) as well as SMX/TMP (2~4-fold), suggesting a stronger and more pleiotropic perturbation of antimicrobial susceptibility. In FOS-selected populations, reduced susceptibility to SMX/TMP (2~4-fold) was consistently observed, representing a distinct but limited collateral effect. Detailed MIC values are provided in Table S4.

### 3.2. Alterations in yghB Contribute to Amphenicol Sensitivity in MEM-R Populations

Carbapenems remain the last-resort treatment for infections caused by ESBL-producing *Enterobacteriaceae*; however, increasing carbapenem resistance has limited their clinical effectiveness [29]. Accordingly, MEM-resistant populations were chosen for further WGS and mechanistic analysis.

**Figure 2:**
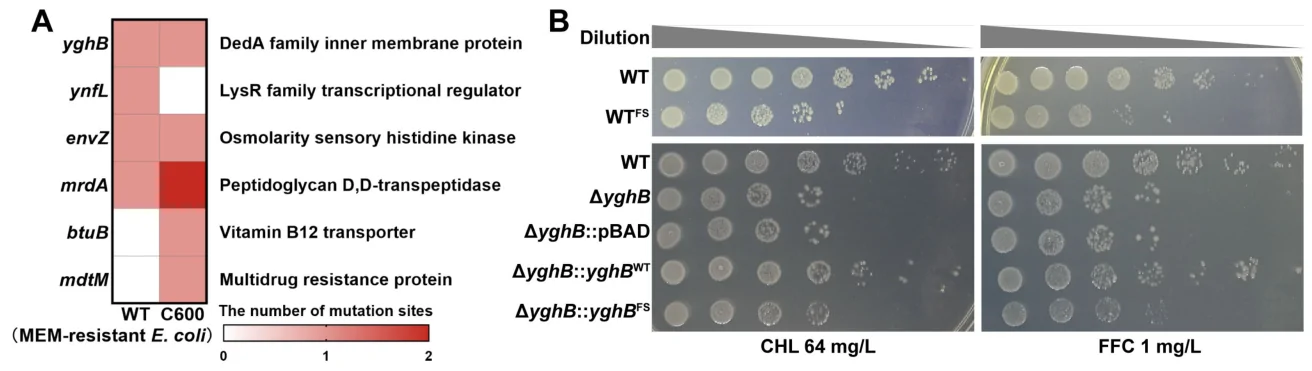
The genetic contributions revealing *yghB*-mediated sensitivity to amphenicols in MEM-R mutants. (**A**) Convergent mutations identified in three parallel MEM-resistant mutants. Color intensity indicates the number of distinct mutation sites detected within each gene. (**B**) Spot assay showing growth of the WT, WT^FS^, Δ*yghB* and complemented strains Δ*yghB*::*yghB*^WT^ and Δ*yghB*::*yghB*^FS^ on MH agar plates containing 64 mg/L CHL or 1 mg/L FFC after overnight incubation at 37 °C. Detailed strain information is provided in Table S1.

Among all genetic variants detected by WGS, we focused on non-synonymous mutations and frameshift indels that were consistently present in independently evolved MEM-resistant mutants. Comparative analysis of WT- and C600-derived lineages identified three recurrent mutational targets, namely *yghB* (encoding a DedA family inner membrane protein), *envZ* (encoding an osmolarity sensor histidine kinase) and *mrdA* (encoding a penicillin-binding protein 2) (Figure 2A). Notably, while mutations repeatedly occurred in the same genetic targets, the specific mutation sites varied among WT- and C600-derived lineages, indicating convergent evolution at the gene level rather than at the nucleotide level (Table S5).

In addition to these shared mutations, background-specific mutations were also observed. WT-derived populations consistently carried a mutation in *ynfL* (encoding a LysR family transcriptional regulator), whereas C600-derived populations harbored mutations in *btuB* (encoding a vitamin B12 transporter) and *mdtM* (encoding a multidrug resistance protein), respectively (Figure 2A). Given that the WT-derived lineage (MEM-R) exhibited the strongest and most reproducible amphenicol-associated collateral sensitivity phenotype, subsequent analyses focused on the MEM-R evolutionary trajectory and the contribution of mutations to the observed phenotype.

Previous studies have implicated both MrdA and EnvZ in carbapenem resistance, prompting us to investigate their contribution to the evolved MEM-resistant phenotype. Among the mutations identified in MEM-R, *mrdA* carried a SNP (G1802A) resulting in a glycine-to-aspartate substitution at residue 601 (MrdA-G601D; Table S5), whereas *envZ* harbored a missense mutation causing an R234C substitution (Table S5). Introduction of either *mrdA* mutant (WT::*mrdA*^G601D^) or *envZ* mutant (WT::*envZ*^R234C^) individually resulted in approximately 2-fold increases in MEM MIC. When combined, the two mutations exhibited an additive effect, increasing the MEM MIC from 0.06 mg/L to 0.2 mg/L (4-fold), supporting their contribution to MEM adaptation.

While *mrdA* and *envZ* contributed to the evolved MEM resistance, they did not affect susceptibility to CHL or FFC. To identify genetic determinants underlying this sensitivity phenotype, we constructed single-gene deletion mutants (Δ*envZ*, Δ*ynfL* and Δ*yghB*) in the WT background, as well as double mutants (Δ*envZ*Δ*yghB* and Δ*ynfL*Δ*yghB*) (Table S1). Among the single deletions, Δ*envZ* and Δ*ynfL* exhibited only minor and antibiotic-specific decreases in MICs (2-fold), with Δ*envZ* affecting SMX/TMP and Δ*ynfL* affecting amphenicols, all within the expected experimental variability (Table 2). In contrast, Δ*yghB* showed a pronounced reduction in amphenicol resistance, with an 8-fold decrease to CHL (from 256 mg/L to 32 mg/L) and FFC (from 4 mg/L to 0.5 mg/L), while having only a minor effect on SMX/TMP susceptibility (2-fold) (Table 2). Importantly, Δ*yghB* did not alter MEM susceptibility, indicating that *yghB* is not involved in MEM resistance but specifically modulates amphenicol sensitivity. Consistent with this, double mutants Δ*envZ*Δ*yghB* and Δ*ynfL*Δ*yghB* exhibited similar or moderately enhanced collateral sensitivity phenotypes compared to Δ*yghB* alone. These included a 4-fold increase in susceptibility to amphenicols and aminoglycosides (APR and AMK; Table 2). Additionally, the Δ*envZ*Δ*yghB* mutant displayed an 8-fold increase in susceptibility to SMX/TMP (from 10 mg/L down to 1.2 mg/L; Table 2). These altered susceptibility profiles, relative to the corresponding single mutants, suggest that combined mutations broaden the collateral phenotypes observed in MEM-R mutants.

To directly determine whether the naturally evolved *yghB* frameshift mutation contributes to amphenicol sensitivity, we reconstructed the chromosomal *yghB* A645del allele in the parental WT background (WT^FS^). The resulting WT^FS^ strain exhibited an 8-fold reduction in MICs to CHL (from 256 mg/L to 32 mg/L) and FFC (from 4 mg/L to 0.5 mg/L), closely recapitulating the phenotype observed in the evolved MEM-R population and Δ*yghB* mutant. To visually reinforce these phenotypes, we performed spot assays on LB agar plates supplemented with CHL or FFC (Figure 2B). Consistent with MIC determination, WT^FS^ showed impaired growth compared with WT under amphenicol exposure. To further establish that this phenotype was associated with loss of YghB function, we complemented the Δ*yghB* strain with pBAD carrying either the wild-type *yghB* or the evolved *yghB* frameshift variant, generating the Δ*yghB*::*yghB*^WT^ and Δ*yghB*::*yghB*^FS^ strains, respectively. Similar to Δ*yghB* and WT^FS^, Δ*yghB*::*yghB*^FS^ exhibited an 8-fold reduction in MICs to CHL (from 256 mg/L to 32 mg/L) and FFC (from 4 mg/L to 0.5 mg/L) relative to the WT strain. Conversely, complementation with wild-type *yghB* (Δ*yghB*::*yghB*^WT^) restored amphenicol resistance to levels comparable with the parental strain (CHL, 256 mg/L; FFC, 4 mg/L). Consistent with these results, spot assays showed impaired growth of Δ*yghB* and Δ*yghB*::*yghB*^FS^ under amphenicol exposure, whereas Δ*yghB*::*yghB*^WT^ restored growth (Figure 2B).Collectively, reconstruction of the evolved chromosomal allele and complementation analysis provide direct genetic evidence that *yghB* disruption is a major contributor to the amphenicol hypersensitivity associated with MEM-R mutants.

### 3.3. The Frameshift Mutation Alters the YghB Structure

**Figure 3:**
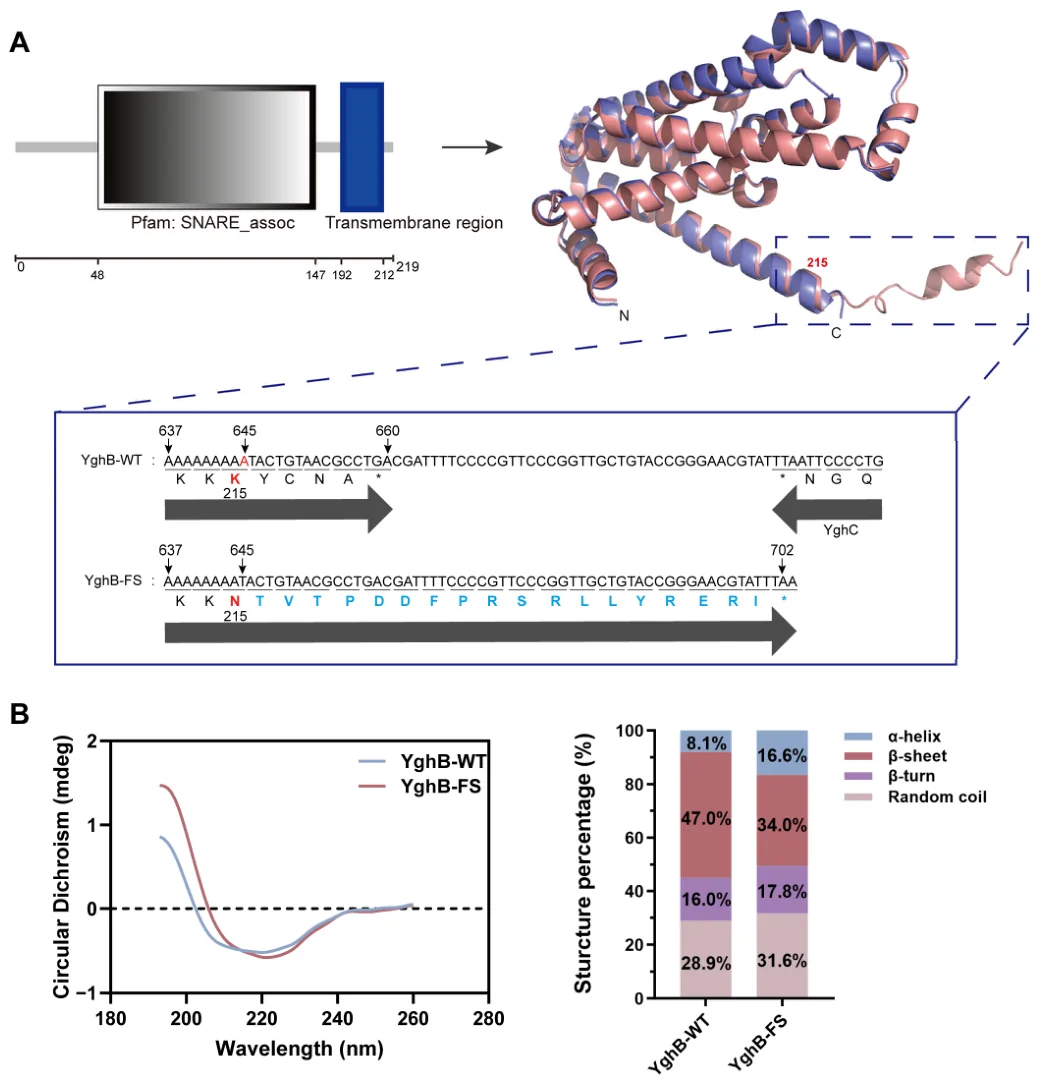
The frameshift mutation alters the structure and secondary conformation of YghB. (**A**) Domain organization and predicted structural models of YghB-WT (purple) and YghB-FS (pink). Red characters indicate the mutation site, and blue characters denote the predicted amino acid residues generated by the frameshift. Structure reference number: P0AA60 (UniProt). This mutation results in overlap of the stop codons of *yghB* and the downstream *yqhC* gene. Asterisks (*) indicate stop codons. (**B**) Circular dichroism of spectra of purified His-tagged YghB-WT and YghB-FS proteins. Spectra represent the averages of three independent experiments, each analyzed in technical triplicates. The accompanying bar chart summarizes the secondary structure composition, showing the alteration in α-helical content and β-sheet content of YghB-FS compared with YghB-WT.

Unlike other nonsynonymous SNPs, the frameshift deletion in *yghB* gene (A645Del) extends the coding sequence (CDS) from 219 to 234 amino acids, which could markedly affect YghB function (Figure 3A). Structural modeling revealed that this mutation occurs within the topological domain (residues 213 to 219), and was predicted to introduce an additional α-helix in the intracytoplasmic C-terminal tail region (Figure 3A). His-tagged YghB-WT and YghB-FS proteins were purified from *E. coli* expression vectors, and their secondary structure was analyzed by circular dichroism CD spectroscopy. The CD analysis indicated an increase in α-helical content (from 8.1% to 16.6%) and a decrease in β-sheet content (from 47.7% to 34%) of YghB-FS compared with YghB-WT (Figure 3B). Although the magnitude of change is relatively modest, combining these CD results with structural modeling suggests that the *yghB* mutation induces conformational changes that may compromise YghB activity or stability.

### 3.4. Disruption of Membrane Permeability and PMF Homeostasis by yghB Mutation Enhances Amphenicols Intracellular Accumulation

Amphenicols exert their antibacterial effect by binding to the 50S ribosomal subunit within the cytoplasm [30]. Thus, efficient cellular uptake is essential for their activity. We hypothesized that the observed collateral sensitivity to amphenicols in MEM-R arises from enhanced intracellular antibiotic accumulation.

**Figure 4:**
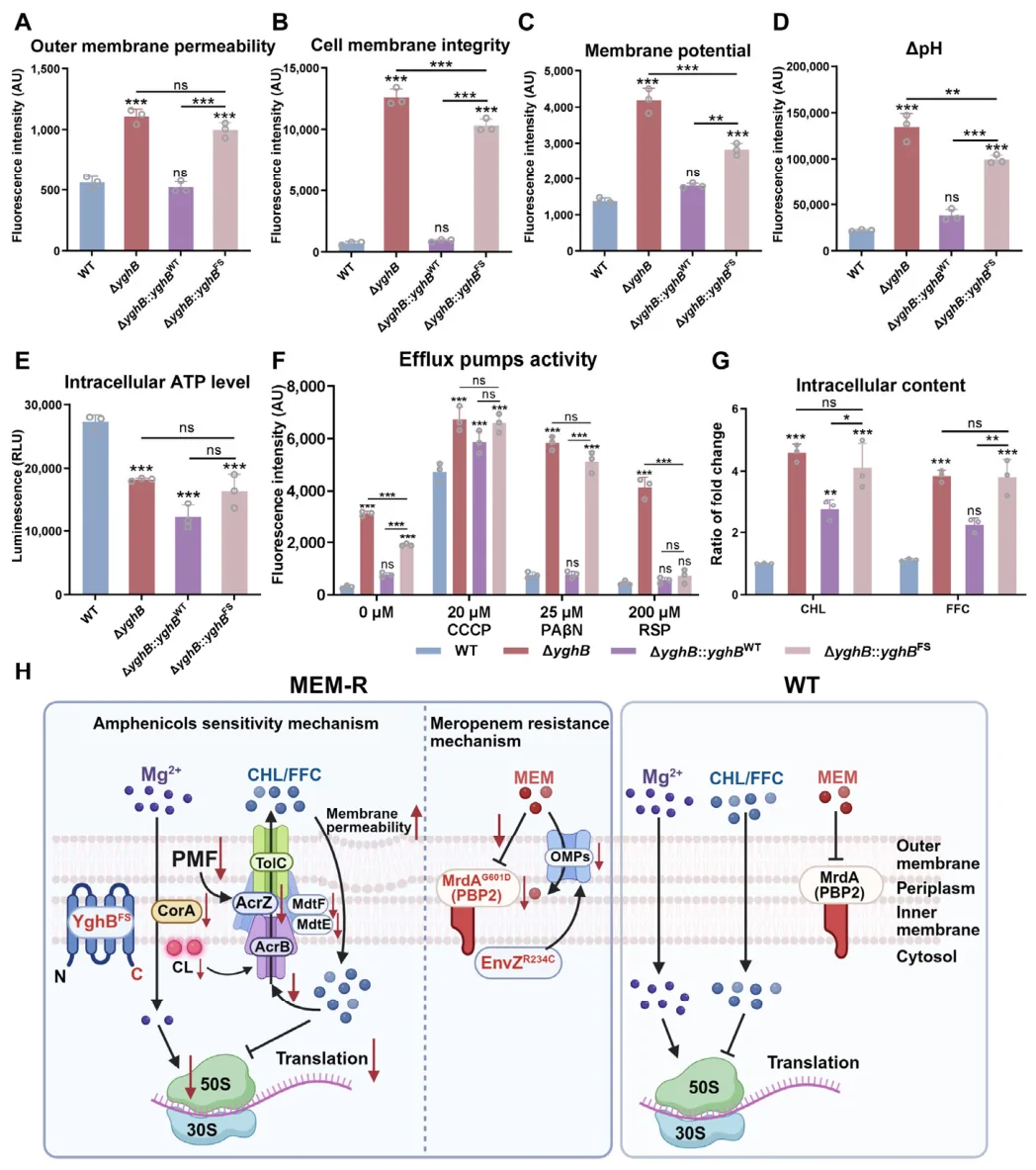
Disruption of membrane integrity and PMF-driven efflux by *yghB* mutation enhances intracellular amphenicols accumulation. (**A**,**B**) The *yghB* mutation increases membrane permeability and compromises membrane integrity compared with the WT strain, as indicated by elevated NPN (**A**) and PI (**B**) fluorescence intensities, respectively. (**C**,**D**) Loss of YghB function perturbs PMF homeostasis compared with the WT strain, as indicated by altered Δψ measured by DiSC_3_(5) fluorescence (**C**) and changes in ΔpH measured by BCECF-AM fluorescence (**D**). (**E**) Loss of YghB function alters cellular energy status, as indicated by reduced intracellular ATP levels in the Δ*yghB* mutant compared with the WT strain. (**F**) Efflux pump activity was assessed by EtBr accumulation assays in the presence or absence of efflux inhibitors (CCCP, PAβN, and RSP). (**G**) Increased intracellular accumulation of amphenicols (CHL and FFC) in *yghB*-deficient strains, determined using LC-MS/MS quantification. (**H**) Schematic representation of the proposed mechanism underlying collateral sensitivity to amphenicols in MEM-R. Resistance acquisition and collateral sensitivity are mechanistically distinct but coexist within the evolved MEM-R background. Created in https://BioRender.com. Data are presented as mean ± SD from three independent biological replicates. CL: cardiolipin. *** *p* < 0.001, ** *p* < 0.01, * *p* < 0.05 and ns *p* > 0.05.

To test this hypothesis, we evaluated membrane permeability, integrity, and PMF in the *yghB* deletion and mutant-complemented strains. As expected, outer membrane permeability (NPN uptake) and plasma membrane integrity (PI staining) were markedly altered in both the Δ*yghB* and Δ*yghB*::*yghB*^FS^ strains, showing significantly increased permeability and compromised integrity compared to the WT strain (*p* < 0.001; Figure 4A,B). These membrane defects caused by the *yghB* mutation were accompanied by alterations in the PMF, which is generated by the membrane potential (Δψ) and the transmembrane proton gradient (ΔpH) across the cytoplasmic membrane. Specifically, both Δ*yghB* and Δ*yghB*::*yghB*^FS^ strains exhibited a substantial reduction in Δψ and an alteration in ΔpH (*p* < 0.01; Figure 4C,D), indicating that YghB deficiency disrupts PMF maintenance and membrane energetics. Complementation with *yghB*^WT^ restored Δψ to near-WT levels and partially recovered ΔpH, confirming that impaired YghB function contributes to PMF destabilization.

Given the observed disruption of PMF homeostasis, we further assessed whether YghB deficiency was associated with broader alterations in cellular energy status by measuring intracellular ATP levels. The Δ*yghB* mutant exhibited significantly reduced ATP levels compared with the parental strain (*p* < 0.001; Figure 4E). However, neither Δ*yghB*::*yghB*^WT^ nor Δ*yghB*::*yghB*^FS^ strains restored ATP levels, suggesting that the altered ATP state may reflect broader physiological consequences associated with YghB disruption or adaptation rather than a direct effect of the *yghB* allele alone.

YghB, a member of the DedA family of membrane proteins, has been proposed to influence the activity of PMF-dependent efflux systems, including the AcrAB-TolC complex [31,32]. Because multiple efflux systems contribute to PMF-dependent antimicrobial extrusion, EtBr accumulation assays combined with efflux inhibitors were used to evaluate overall efflux capacity. Therefore, we quantified EtBr accumulation in the WT, Δ*yghB*, and complemented strains, with and without efflux inhibitors such as CCCP, PAβN and RSP. Compared to WT and Δ*yghB*::*yghB*^WT^, both Δ*yghB* and Δ*yghB*::*yghB*^FS^ strains exhibited significantly elevated EtBr fluorescence, indicative of reduced efflux-associated activity, especially in the presence of PMF disruptors CCCP (Figure 4F). These results indicate that YghB disruption is associated with reduced PMF-dependent efflux capacity, which may contribute to impaired extrusion of antimicrobial compounds, although the contribution of individual efflux systems cannot be distinguished by EtBr assays alone.

Consistent with altered efflux-associated activity and membrane perturbation, intracellular accumulation of CHL and FFC increased by 4.6- and 3.8-fold, respectively, in the Δ*yghB* mutant relative to WT (*p* < 0.01; Figure 4G). Similar results were observed in Δ*yghB*::*yghB*^FS^ compared with Δ*yghB*::*yghB*^WT^ (*p* < 0.01; Figure 4G). To further evaluate whether *yghB* alteration was associated with broader physiological effects beyond membrane-associated phenotypes, we analyzed growth characteristics under non-selection conditions. Compared with the WT strain, the Δ*yghB* mutant exhibited reduced maximum growth rate and decreased area under the growth curve (AUC), whereas the lag phase duration remained largely unchanged (Figure S1). The Δ*yghB*::*yghB*^WT^ strain displayed partially improved growth performance compared with the Δ*yghB* mutant, with an increased AUC, whereas the Δ*yghB*::*yghB*^FS^ strain showed altered growth characteristics, including a lower AUC and prolonged lag phase. These results suggest that *yghB* alteration affects cellular physiological homeostasis, consistent with its role in maintaining membrane-associated functions. Together, these findings demonstrate that *yghB* alteration is associated with altered membrane barrier properties, PMF homeostasis and reduced efflux-associated activity, collectively contributing to increased intracellular accumulation of amphenicols. This multifactorial alteration underlies the sensitivity of MEM-R to amphenicols (Figure 4H).

### 3.5. Transcriptomic Analyses and Functional Characterization Reveal Mechanisms Associated with Amphenicol Sensitivity

#### 3.5.1. Transcriptomic Profiling Reveals Physiological Remodeling During MEM Adaptation

Transcriptomic profiling was performed to characterize the physiological changes associated with MEM resistance evolution. KEGG enrichment analysis revealed that differentially expressed genes were mainly enriched in phosphotransferase systems (PTS), oxidative phosphorylation and metabolic pathways (Figure S2A). Consistent with the enrichment of membrane-associated pathways, several genes involved in outer membrane permeability and efflux-associated functions were differentially expressed, including decreased expression of outer membrane proteins *ompF*, *ompN* and *ompW* and altered expression of efflux-associated genes such as *acrZ* and *mdtEF* (Figure S2B). In addition, multiple genes associated with energy metabolism were affected, including downregulation of cytochrome oxidase genes *cydAB* and *cyoABCD* and several PTS-related genes, suggesting transcriptional remodeling of energy metabolism during MEM adaptation (Figure S2B). Collectively, transcriptomic analysis of MEM-R mutants revealed broad remodeling of membrane-associated functions, transport systems and energy metabolism during meropenem adaptation. These transcriptomic changes characterize the global physiological remodeling associated with MEM adaptation. However, because evolved MEM-R isolates harbor multiple genetic alterations, the contribution of individual recurrent mutations cannot be directly resolved from the evolved transcriptome alone. Therefore, we further investigated the specific functional consequences of the recurrent *yghB* mutation using an isogenic Δ*yghB* mutant.

#### 3.5.2. Loss of YghB Function Is Associated with Mg^2+^ Limitation and Altered Ribosome-Related Processes

**Figure 5:**
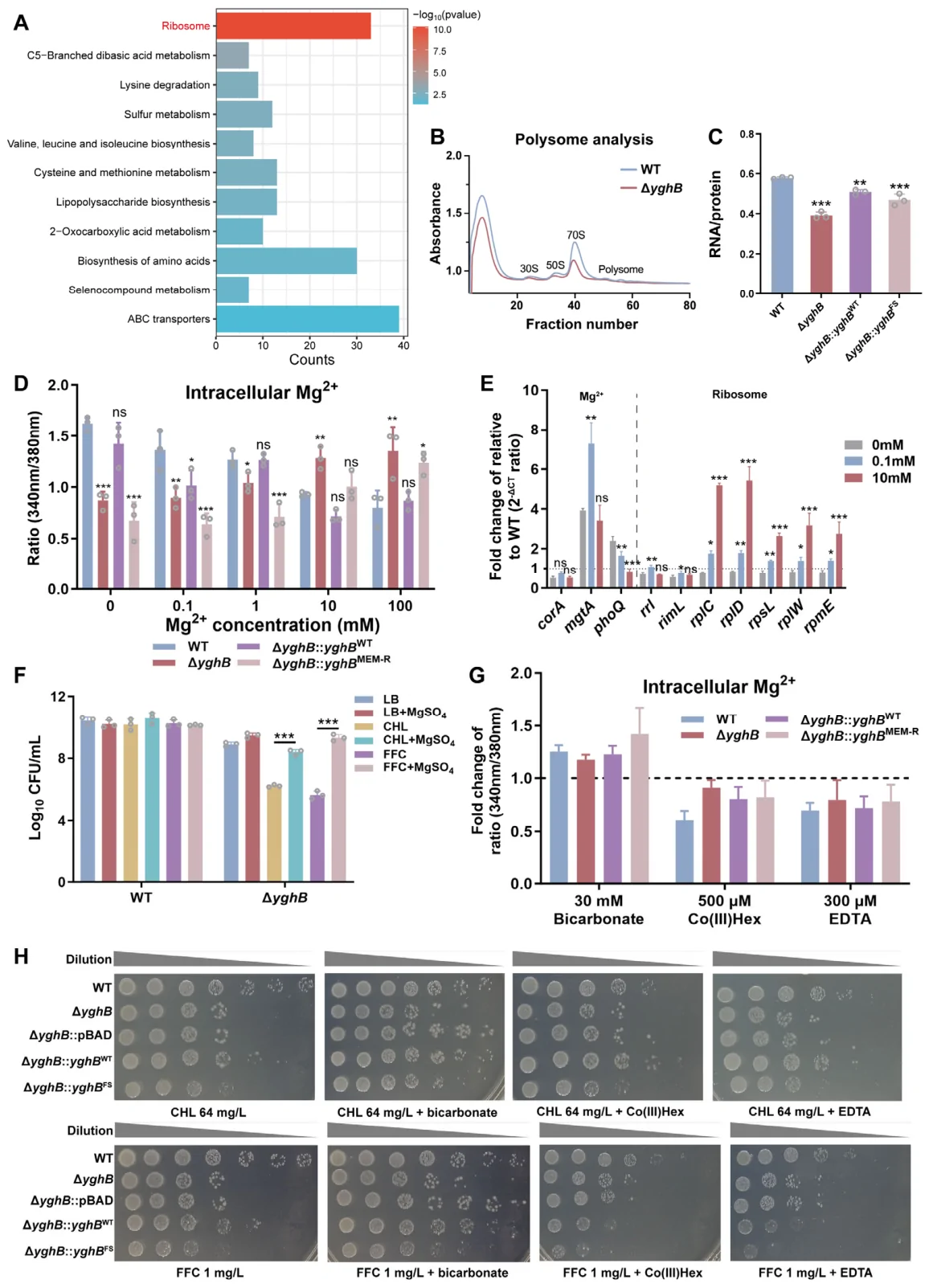
The *yghB* mutation disrupts Mg^2+^ homeostasis and ribosome homeostasis, promoting collateral sensitivity to amphenicols. (**A**) KEGG enrichment analysis of differentially expressed genes in the Δ*yghB* mutant relative to the WT. (**B**) Polysome profiling showing reduced 70S ribosome abundance in the Δ*yghB* mutant compared with the WT. (**C**) RNA/protein ratio of the WT, Δ*yghB* and its complemented strains, reflecting translational capacity. (**D**) Intracellular Mg^2+^ levels in the WT, Δ*yghB* and complemented strains grown in standard LB medium with or without Mg^2+^ supplementation. (**E**) Relative transcriptional levels of Mg^2+^ transporters and ribosome-associated genes in WT and Δ*yghB* strains grown in standard LB medium supplemented with different concentrations of Mg^2+^. Expression values were calculated as 2^−∆CT^ for each strain and are presented as the ratio 2^−∆CT^_Δ*yghB*_/2^−∆CT^_WT_ at the corresponding Mg^2+^ concentration. (**F**) Effect of Mg^2+^ supplementation (50 mM) in standard LB medium on the amphenicol sensitivity of WT and Δ*yghB* strains, as assessed by growth in the presence or absence of CHL (128 mg/L) or FFC (2 mg/L). (**G**,**H**) Effect of bicarbonate (PMF modulator), Co(III)Hex (CorA inhibitor), and EDTA (Mg^2+^ chelator) on intracellular Mg^2+^ concentrations (**G**) and amphenicol sensitivity (**H**) in WT, Δ*yghB* and complemented strains grown under standard LB conditions. *** *p* < 0.001, ** *p* < 0.01, * *p* < 0.05 and ns *p* > 0.05. Unless otherwise stated, data are presented as mean ± SD from three independent biological replicates.

Comparative transcriptomic analysis between the ΔyghB mutant and its parental strain revealed significant downregulation of genes involved in ribosomal structure, translation, and protein synthesis (Figure 5A). In particular, genes encoding chloramphenicol-binding ribosomal proteins, such as *rplC*, *rplD*, and *rpsL*, were significantly suppressed (Figure S3), suggesting altered ribosome biogenesis and translation-related processes. Given that ribosomal stability and translational accuracy critically depend on intracellular Mg^2+^ levels [26,33], these transcriptional changes suggest that loss of YghB function may affect Mg^2+^-dependent ribosome-associated processes.

Polysome profiling further confirmed a pronounced reduction in 70S ribosome abundance in Δ*yghB* relative to the WT (Figure 5B), consistent with altered ribosome assembly under Mg^2+^ deficient conditions. Correspondingly, the RNA/protein ratio, a proxy for translational capacity, was significantly lower in both Δ*yghB* and its complement Δ*yghB*::*yghB*^FS^ (Figure 5C), suggesting reduced translational capacity and altered ribosome activity. As amphenicols exert their antibacterial action by binding to ribosomal subunits [30], impaired ribosome-associated processes in the Δ*yghB* background may increase susceptibility by reducing cellular tolerance to ribosome-targeting antibiotics.

In addition, broad downregulation of inner membrane proteins (*yagU*, *yaiY*, *ycdZ*, *ydgK*, *yhiM*, *yhjD*, *yjiG* and *yqjE*) and cyclopropane fatty acid synthase (CFAS) suggested altered membrane-associated functions, which may influence Mg^2+^ homeostasis (Figure S3). Reduced expression of *osmY* and *otsAB*, involved in osmotic stress tolerance, further supports reduced membrane homeostasis. Conversely, the upregulation of waa cluster genes (*waaB*, *waaO*, *waaU*, *waaZ*; Figure S3), which mediate LPS core truncation [34], may alter outer membrane permeability and amplify the physiological consequences of PMF dissipation. Regarding PMF-dependent efflux-associated systems, transcriptomic analysis revealed significant downregulation of *acrZ*, *mdtE* and *mdtF*, whereas no significant changes were detected in the transcriptional levels of the core efflux genes (e.g., *acrAB*, *tolC*) (Figure S3). These results suggest that YghB disruption may influence efflux-associated functions through alterations in accessory factors (e.g., *acrZ*) and alternative efflux systems (e.g., *mdtEF*), rather than through transcriptional repression of the core *acrAB* and *tolC* genes.

#### 3.5.3. Mg^2+^ Supplementation Restores Ribosome-Associated Processes and Partially Rescues Amphenicol Resistance

The two-component system PhoQ/PhoP senses periplasmic Mg^2+^ levels and activates mgtA transcription in response to Mg^2+^ limitation [35,36]. Transcriptomic profiling revealed significant upregulation of *mgtA* and *phoQ* in the Δ*yghB* mutant (Figure S3), suggesting compensatory activation of Mg^2+^ uptake machinery under cytoplasmic Mg^2+^ depletion. To determine whether loss of YghB function affects Mg^2+^ homeostasis, intracellular Mg^2+^ levels were first quantified under standard LB growth conditions without additional Mg^2+^ supplementation. Compared with the WT strain, Δ*yghB* and Δ*yghB*::*yghB*^FS^ exhibited reduced intracellular Mg^2+^ levels under basal conditions (Figure 5D), indicating impaired Mg^2+^ homeostasis associated with *yghB* disruption. We next examined whether altered extracellular Mg^2+^ availability could influence intracellular Mg^2+^ accumulation. When exposed to increasing extracellular Mg^2+^ concentrations, both Δ*yghB* and Δ*yghB*::*yghB*^FS^ strains accumulated Mg^2+^ more rapidly than the WT strain in a concentration-dependent manner (Figure 5D), suggesting an enhanced compensatory response to extracellular Mg^2+^ availability in the context of impaired intracellular Mg^2+^ homeostasis.

Consistent with the restoration of intracellular Mg^2+^ availability, Mg^2+^ supplementation reactivated the expression of ribosome-associated genes that were suppressed in Δ*yghB*, while *phoQ* expression declined in a concentration-dependent manner (Figure 5E), reflecting the reestablishment of Mg^2+^ sufficiency. Expression of *mgtA* peaked at 0.1 mM Mg^2+^ but was suppressed at high Mg^2+^ levels (10 mM), consistent with feedback regulation of Mg^2+^ transport [35]. In contrast, CorA, another primary Mg^2+^ transporter in *E. coli* [35], exhibited minimal transcriptional variation across Mg^2+^ concentrations (Figure 5E). These findings suggest that restoration of Mg^2+^ homeostasis contributes to the recovery of ribosome-associated gene expression and may improve translational capacity.

To determine whether restoration of Mg^2+^ homeostasis influences antibiotic susceptibility, we next examined the response of Δ*yghB* to amphenicols in the presence of exogenous Mg^2+^. As expected, Mg^2+^ supplementation partially restored resistance to CHL and FFC in Δ*yghB* (Figure 5F), indicating that disruption of Mg^2+^ homeostasis contributes to *yghB*-associated amphenicol sensitivity. To further investigate the underlying mechanism, we examined the effects of Mg^2+^ on PMF and membrane function. Elevated extracellular Mg^2+^ further reduced Δψ in the Δ*yghB* mutant (Figure S4A). In contrast, it partially restored membrane integrity (Figure S4B,C), consistent with its established role in stabilizing the bacterial envelope.

#### 3.5.4. PMF-Dependent Mg^2+^ Influx Contributes to *yghB*-Associated Amphenicol Sensitivity

Mg^2+^ transport in *E. coli* relies on the Δψ component of PMF [37]. Given that loss of YghB function was associated with reduced Δψ, we hypothesized that altered PMF status may compromise Mg^2+^ uptake [38]. Consistent with this, bicarbonate supplementation, which collapses the ΔpH component while compensatorily enhancing Δψ (Figure S5), significantly increased intracellular Mg^2+^ levels and partially restored resistance to chloramphenicol and florfenicol in both Δ*yghB* and Δ*yghB*::*yghB*^FS^ strains (Figure 5G,H). Conversely, inhibition of CorA Mg^2+^ transporter with Co(III)Hex or chelation of Mg^2+^ by EDTA markedly reduced intracellular Mg^2+^ levels and further sensitized *E. coli* to amphenicols, especially in the wild-type background (Figure 5H).

Collectively, these findings support a model (Figure 4H) in which loss of YghB function alters PMF-associated processes, leading to reduced Δψ and impaired Mg^2+^ influx. Mg^2+^ limitation is associated with impaired ribosome assembly and reduced ribosome-associated translational capacity, which may enhance the inhibitory effects of ribosome-targeting amphenicols. In addition, altered membrane-associated functions and efflux-associated activity may contribute to increased intracellular antibiotic accumulation. Together, these findings indicate that YghB disruption represents one mechanism contributing to amphenicol sensitivity observed in MEM-R mutants.

### 3.6. Collateral Sensitivity-Based Therapeutic Potential Against MEM-R Mutants In Vitro and In Vivo

**Figure 6:**
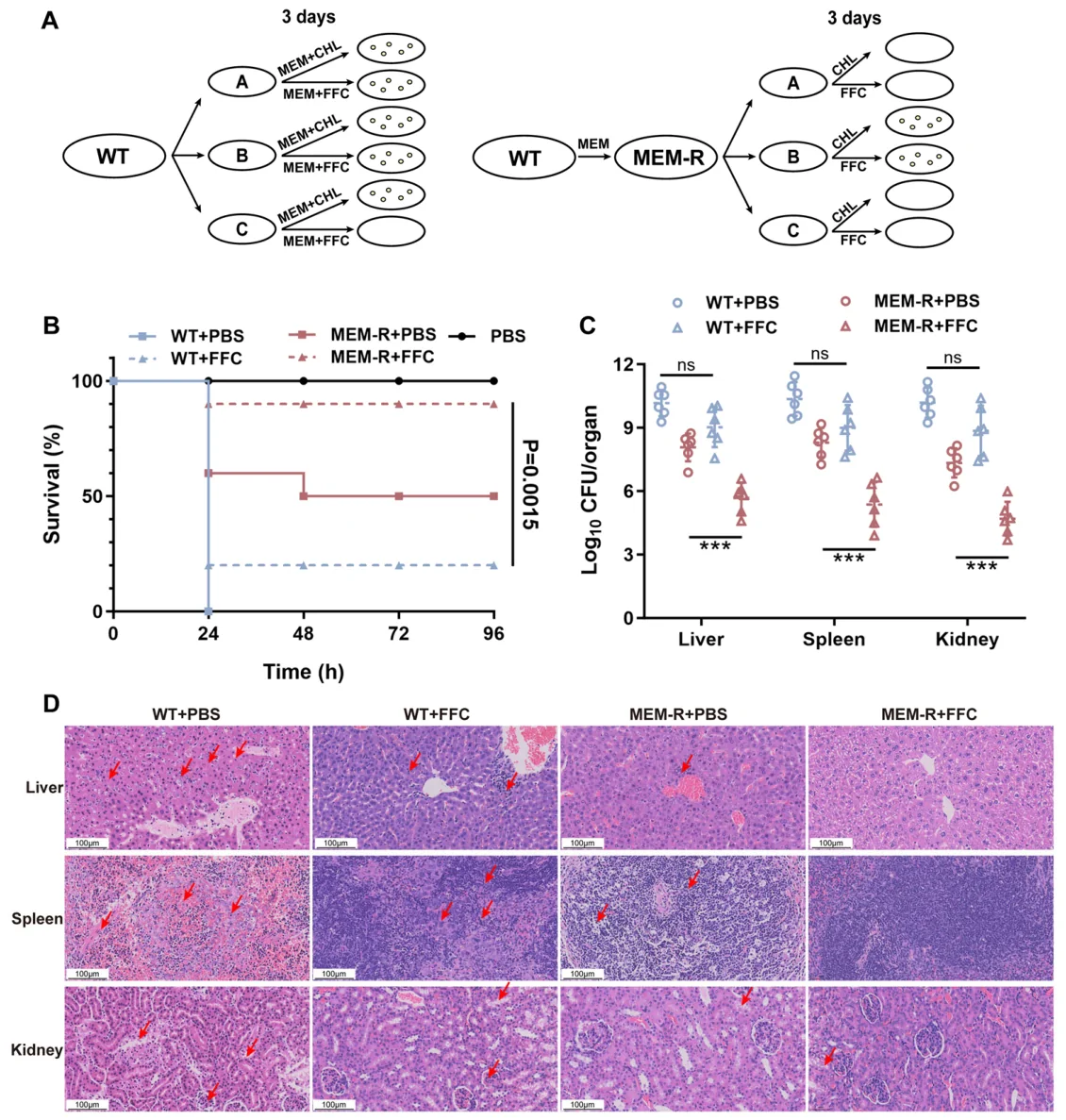
Collateral sensitivity-based therapeutic efficacy against MEM-R mutants in vitro and in vivo. (**A**) In vitro population survival of WT and MEM-R under antibiotic exposure. WT populations were subjected to combined meropenem and amphenicol treatment, whereas MEM-R populations were propagated in the presence of CHL or FFC alone. (**B**) Survival curve of *G. mellonella* larvae infected with the WT or MEM-R strains and treated with different antibiotic regimens (*n* = 10 per group). (**C**) Bacterial burdens in the liver, spleen, and kidney of mice infected with WT or MEM-R strains following various treatments. Data are presented as mean ± SD. *** *p* < 0.001 and ns *p* > 0.05. (**D**) Representative H&E-stained sections of liver, spleen and kidney tissues from mice infected with the WT or MEM-R strain and treated with FFC or PBS. FFC treatment markedly alleviated tissue damage in MEM-R infections. Red arrows indicate representative histopathological lesions, including hepatocellular degeneration and necrosis, lymphoid depletion and necrotic lesions in the spleen, and renal tubular injury or lipid accumulation in the kidney.

To evaluate whether the collateral sensitivity of MEM-resistant *E. coli* to amphenicols could be leveraged as a potential therapeutic strategy, we evaluated collateral sensitivity-based intervention strategies using MEM and amphenicols in vitro and in vivo. In vitro, the WT populations were exposed to combined MEM and amphenicol treatment, whereas parallel MEM-R populations were exposed to CHL or FFC treatment alone (Figure 6A). After 3 days of treatment, nearly all WT populations survived the combined treatment, whereas only two of six MEM-R populations remained viable, indicating that amphenicol treatment effectively eradicated resistant lineages by exploiting collateral sensitivity (Figure 6A).

We next evaluated the therapeutic potential of exploiting collateral sensitivity in vivo using *G. mellonella* and murine peritonitis-septicemia infection models. Although MEM-R populations exhibited collateral sensitivity to both CHL and FFC in vitro, FFC was selected for subsequent in vivo evaluation because CHL use is restricted in many countries due to toxicity concerns, whereas FFC has an established history of use in veterinary medicine. In the *G. mellonella* model, MEM-R exhibited attenuated virulence compared with the parental WT strain, as all larvae infected with WT died within 24 h, whereas 50% of larvae infected with MEM-R survived up to 96 h (Figure 6B). Following FFC treatment, survival of MEM-R-infected larvae further increased to 90%, representing a significantly greater therapeutic benefit than that observed in WT-infected larvae (*p* = 0.0015; Figure 6B).

Consistent with the attenuated virulence observed in *G. mellonella*, MEM-R-infected mice harbored 2.06~2.86 log10 cfu/g fewer bacteria in the liver, spleen, and kidney than mice infected with the WT strain (Figure 6C). Importantly, FFC treatment produced substantially greater bacterial clearance in MEM-R infections, reducing organ burdens by 3.36~4.16 log10 CFU/g, whereas reductions in WT infections were less than 1.2 log10 CFU/g (*p* < 0.001; Figure 6C). Histopathological analysis further showed that MEM-R infection caused milder tissue damage than WT infection, and that FFC treatment markedly alleviated pathological lesions in the livers, spleens, and kidneys (Figure 6D). Together, these findings indicate that meropenem resistance evolution is associated with both attenuated virulence and enhanced susceptibility to FFC treatment, supporting the therapeutic potential of collateral sensitivity-based intervention strategies.

Collectively, these results demonstrate that collateral sensitivity-based interventions can improve treatment outcomes against MEM-R infections in experimental infection models. This evolution-informed therapeutic strategy highlights the potential of exploiting resistance trade-offs to combat multidrug-resistant *E. coli* and other clinically relevant Gram-negative pathogens.

## 4. Discussion

In this study, we identified a robust and conserved collateral sensitivity to amphenicols that repeatedly emerged during antibiotic adaptation in both plasmid-bearing and plasmid-free *E. coli* lineages. Despite marked differences in resistance determinants and evolutionary trajectories, amphenicol hypersensitivity consistently evolved following MEM, APR, and COL selection pressures, suggesting convergence toward common susceptibility constraints. Notably, recent large-scale clinical surveillance analyses revealed that most collateral sensitivity interactions are species-specific and rarely conserved across multiple pathogens, yet CHL participates in several recurrent collateral sensitivity relationships [39]. Collectively, these observations suggest that amphenicols may represent recurrent collateral sensitivity endpoints that can emerge across distinct evolutionary contexts.

Consistent with previous reports, MEM-driven evolution has been associated with collateral sensitivity to multiple antibiotic classes, including cephalosporins, amphenicols, carbapenems, sulfonamides and aminoglycosides [40,41,42]. Notably, MEM exposure in *Burkholderia multivorans* increases susceptibility to CHL and SMX [41,42], while a strong reciprocal collateral sensitivity between imipenem and CHL has been reported in *Streptococcus pneumoniae* [39]. Together with our findings, these observations suggest that carbapenem-associated amphenicol hypersensitivity can emerge across phylogenetically distinct bacterial species. However, the underlying physiological and mechanistic determinants remain poorly understood. Collateral sensitivity patterns associated with APR-driven evolution have been reported across multiple antibiotic classes, including cephalosporins, tetracyclines, polymyxin, sulfonamides and amphenicols [10,11]. In this context, our observations are broadly consistent with previously described APR-associated collateral sensitivity patterns at the level of affected antibiotic classes. Notably, recent clinical surveillance analyses identified conserved collateral sensitivity interactions between colistin resistance and increased susceptibility to cefazolin, ampicillin, and LVF across multiple bacterial species [39]. In contrast, evidence for colistin-associated collateral sensitivity to ribosome-targeting 50S inhibitors remains limited. Our observation that COL adaptation repeatedly promotes amphenicol hypersensitivity therefore expands the known spectrum of COL-associated collateral sensitivity. Together, these observations support the notion that amphenicols may constitute recurrent evolutionary endpoints of collateral sensitivity.

In addition, we observed broad-spectrum resensitization to cephalosporins (CTX and CEF) in evolved meropenem-resistant mutants. Unlike previously reported CTX-M-dependent resensitization [43], neither mutations in *bla*_CTX-M-55_ nor loss of the associated plasmid were detected in our mutants. The increased susceptibility to cephalosporins observed in the MEM-R mutants is likely attributable to multiple interconnected physiological changes. Transcriptomic analysis between MEM-R mutants and its parental strain revealed differential expression of genes associated with membrane permeability and efflux-associated functions. Specifically, downregulation of outer membrane proteins (OmpF, OmpN and OmpW) [44] and multidrug efflux proteins (AcrZ and MdtEF) [45,46] may alter intracellular cephalosporin exposure and potentially contribute to increased accumulation (Figure S2). In addition, reduced expression of genes involved in carbohydrate transport PTS (e.g., *agaVW*, *gatABC*, *manXYZ*, *pstA*, *srlABE*) [47] and cytochrome oxidase genes (*cydAB* and *cyoABCD*) [48] suggests remodeling of energy metabolism during MEM adaptation (Figure S2). Collectively, the evolved transcriptional remodeling observed in MEM-R mutants may contribute to increased intracellular cephalosporin exposure and enhanced susceptibility.

To gain mechanistic insight into the recurrent amphenicol hypersensitivity observed in our study, we focused on the MEM-evolved populations, for which genomic and transcriptomic data were available. Although several background-specific mutations were detected, including *btuB* and *mdtM* in the C600-derived populations, these alterations were not consistently observed across independently evolved lineages. We therefore focused on mutations associated with resistance and collateral sensitivity phenotypes. Functional SNP analysis suggested that the resistance and sensitivity phenotypes were associated with multiple mutations involving *mrdA*, *envZ*, *ynfL* and *yghB*, each contributing distinct yet interdependent effects on cell envelope physiology. Previous studies have established that mutations in *mrdA* and *envZ* emerge early during MEM selection pressure and are crucial for high-level resistance [49,50]. In particular, EnvZ, as the sensor kinase of the EnvZ/OmpR two-component system, regulates the expression of outer membrane proteins and thereby influences outer membrane permeability and antibiotic influx [51,52]. Alterations in EnvZ-mediated signaling can therefore indirectly affect antibiotic susceptibility by remodeling membrane barrier properties. EnvZ-mediated regulation of outer membrane proteins has been implicated in carbapenem resistance, consistent with reports that high-level meropenem resistance can arise from specific combinations of mutations involving *envZ*, *ftsI*, *mrdA*, *acrB* and *acrR* [50]. Consistent with these findings, our results confirm that *mrdA* and *envZ* mutations are key determinants of MEM resistance, although the specific mutation sites differ from those previously reported [49,50] (Table S5). Importantly, while *yghB* alone confers the strongest sensitivity to CHL and FFC, combined mutations involving *envZ* or *ynfL* broadened susceptibility changes across additional antibiotics. Given the roles of EnvZ-mediated envelope stress responses [51,52] and LysR-type transcriptional regulation in modulating membrane-associated and stress-response pathways, these findings support a multifactorial contribution to collateral sensitivity, in which perturbations of regulatory networks collectively reprogram bacterial susceptibility profiles. Although *yghB* does not appear to play a direct mechanistic role in meropenem resistance, the independent emergence of the same *yghB* frameshift mutation across all MEM-resistant populations suggests a reproducible association with meropenem selection pressure. This repeated emergence suggests a conserved evolutionary linkage with MEM selection pressure, with potential relevance for anticipating evolutionary trajectories under carbapenem selection pressure. However, because the evolved populations contain multiple concurrent mutations, the specific contribution of each individual allele, particularly the evolved *yghB* frameshift variant, remains to be further resolved through precise allele reconstruction. Moreover, the Δ*yghB* strain used for functional characterization represents a complete loss-of-function model, which may produce stronger physiological perturbations than the naturally occurring *yghB* frameshift allele identified during MEM evolution. Therefore, although Δ*yghB* recapitulates the collateral sensitivity phenotype, the magnitude of the associated membrane, PMF, and Mg^2+^ homeostasis defects may not fully reflect the effects of the evolved allele.

Previous studies have identified YghB and other DedA family proteins as conserved inner membrane proteins involved in maintaining membrane homeostasis, ion balance, and cellular energetics [53,54]. In particular, *E. coli* YqjA and YghB have been proposed to function as membrane transport-related proteins, and mutations in these proteins can activate envelope stress responses and impair growth under specific environmental conditions [55,56]. Previous studies have also linked DedA family proteins to antibiotic susceptibility in several bacterial species. In *E. coli*, mutations affecting YghB or YqjA compromise resistance to several toxic compounds and antimicrobial agents, including ethidium bromide, acriflavine, benzalkonium chloride, and methyl viologen, which are substrates of PMF-dependent efflux systems, suggesting that DedA proteins influence antibiotic susceptibility through membrane energetics and transport functions [53,55]. Similarly, DedA family proteins have been implicated in resistance to colistin, a membrane-targeting antibiotic, in *Klebsiella* and *Burkholderia* species [14,31,57,58], further supporting a conserved role of DedA proteins in envelope-associated antibiotic defense. Consistent with these previous observations, our results demonstrate that disruption of *yghB* compromises Δψ and cellular energy status. However, unlike previous studies that mainly characterized DedA family proteins through deletion mutants, stress phenotypes, or intrinsic resistance traits, our findings reveal that YghB dysfunction can emerge during antibiotic adaptation and contribute to an evolutionary trade-off between meropenem resistance and collateral sensitivity. Our findings therefore reveal an unrecognized connection between DedA-mediated membrane physiology and translation-associated antibiotic susceptibility.

Mechanistically, our structural analyses suggest that the *yghB* mutation alters the C-terminal cytoplasmic extension, which shares structural similarity with the osmosensing coiled-coil domain of ProP and may contribute to maintenance of transmembrane potential [59]. Disruption of this region may destabilize the coiled-coil configuration, resulting in proton leakage and dissipation of Δψ. Beyond PMF dissipation, the reduced ATP levels observed following YghB disruption further support an altered cellular energetic state. In bacteria, intracellular ATP availability is determined by the integrated balance between membrane-associated energy generation, proton motive force maintenance, ion homeostasis, and metabolic activity rather than by a single component alone [60]. Therefore, the incomplete restoration of ATP levels after complementation suggests that YghB disruption may trigger broader physiological consequences associated with disturbed membrane energetics, which are not completely reversed by restoration of YghB function alone. Such effects may arise from persistent alterations in membrane-associated processes or downstream metabolic regulation following disruption of cellular homeostasis.

Transcriptomic profiling revealed significant downregulation of *acrZ*, a stabilizing accessory factor of the AcrAB-TolC complex, as well as *mdtE* and *mdtF*, which encode components of the RND-type MdtEF-TolC system [45,46]. AcrZ directly associates with the transmembrane domain of AcrB and modulates its conformational state, thereby altering substrate selectivity and potentially affecting CHL efflux efficiency [45]. Given that cardiolipin deficiency has been shown to increase membrane fluidity and permeability and to exacerbate CHL susceptibility in AcrZ-deficient backgrounds [45,61,62,63]. We observed downregulation of *cls*, encoding cardiolipin synthase, these transcriptional changes collectively suggest that both membrane integrity and efflux-associated functions may be compromised (Figure S6). In parallel, MdtE and MdtF downregulation may further reduce the capacity of an alternative RND-type efflux route for CHL, potentially contributing to intracellular drug accumulation [64,65,66]. Importantly, these alterations occurred without detectable changes in the transcription of *acrAB* and *tolC*, indicating that efflux impairment is not driven by global suppression of the AcrAB-TolC system. Rather, the combined reduction of *acrZ*, *cls* and *mdtEF* suggests a pathway-specific remodeling of envelope-associated antibiotic defense. Together, these changes may contribute to altered CHL efflux-associated processes and increased intracellular retention of CHL.

YghB has been associated with altered antibiotic susceptibility and membrane stress phenotypes when disrupted, and these effects can be partially alleviated by supplementation with divalent cations such as Mg^2+^ or Ca^2+^ [54,61]. Recent studies in *Klebsiella pneumoniae* (*K. pneumoniae*) further demonstrated that DedA family proteins contribute to divalent cation homeostasis and bacterial stress tolerance, highlighting a conserved association between DedA family functions and metal ion regulation [14]. Consistent with these observations, our study shows that YghB disruption perturbs Mg^2+^ homeostasis in *E. coli* and that Mg^2+^ supplementation partially restores amphenicol resistance. These findings extend previous studies of DedA family proteins by linking DedA-associated cation imbalance to antibiotic susceptibility involving ribosome-targeting compounds. However, whether analogous evolutionary mechanisms contribute to collateral sensitivity in other *Enterobacterales* remains to be determined. Although DedA/YghB family proteins share conserved physiological functions related to membrane homeostasis and divalent cation regulation, individual family members exhibit sequence divergence, functional redundancy, and species-specific physiological roles that may influence their contributions to antibiotic adaptation. Therefore, comparative studies across clinically relevant *Enterobacterales* will be required to determine whether DedA/YghB-associated evolutionary trajectories leading to collateral sensitivity are conserved.

Mg^2+^ is essential for ribosome assembly, structural stability and translational fidelity [26,33,67]. Therefore, the partial restoration of amphenicol resistance following Mg^2+^ supplementation likely reflects improved ribosome-associated functions rather than recovery of Δψ [67]. Although PMF dissipation has been proposed as a potential consequence of YghB disruption, our results suggest that altered Mg^2+^ availability represents an additional physiological consequence of YghB dysfunction. Membrane destabilization, altered activity or regulation of Mg^2+^ transport systems, and broader envelope stress responses may contribute to Mg^2+^ imbalance independently of PMF changes [35]. Thus, Mg^2+^ dysregulation likely acts as a secondary factor that amplifies amphenicol sensitivity in combination with PMF dissipation and efflux impairment, rather than representing a direct downstream consequence of Δψ collapse alone. Collectively, our findings extend previous studies of DedA family proteins by demonstrating that YghB is not only involved in membrane homeostasis and antibiotic resistance but can also act as an evolutionary determinant of collateral sensitivity. Through coordinated effects on membrane energetics, efflux capacity, and Mg^2+^-dependent ribosomal function, YghB disruption establishes a mechanistic link between DedA family biology and antibiotic resensitization.

From a therapeutic perspective, our findings demonstrate that amphenicol hypersensitivity can be exploited as an evolutionary vulnerability associated with carbapenem resistance. Despite widespread resistance and the clinical restriction of CHL due to toxicity concerns, amphenicols remain valuable agents in veterinary medicine and provide important models for investigating collateral sensitivity-based therapeutic principles [68,69]. However, the clinical translation of this strategy requires careful consideration. CHL has limited clinical use in many countries, whereas FFC is primarily used in veterinary medicine [70]. Therefore, our findings should be interpreted as establishing the feasibility of targeting evolutionary trade-offs associated with carbapenem resistance rather than supporting immediate clinical application of these specific agents. Future studies will be required to determine whether similar collateral sensitivity-based strategies can be leveraged using clinically applicable ribosome-targeting compounds.

While this study provides mechanistic insight into amphenicol hypersensitivity in MEM-evolved populations, the mechanistic basis of collateral sensitivity observed in APR- and COL-evolved lineages was not further investigated. Furthermore, although genetic and functional analyses support *yghB* as a key contributor to MEM-associated collateral sensitivity, the evolved MEM-resistant populations harbor additional adaptive mutations. Therefore, precise reconstruction of the evolved *yghB* frameshift allele in the parental chromosome will be required to disentangle its allele-specific contribution and validate its physiological effects in the original genetic context. In addition, although our work demonstrates that collateral sensitivity-based interventions can improve treatment outcomes against MEM-resistant infections in experimental models, the in vivo experiments did not implement dynamic antibiotic cycling or alternating regimens. Resistance evolution in vivo is governed by complex and heterogeneous selection pressures, including host immune responses, spatial and temporal drug gradients, and fitness trade-offs in virulence and metabolism. Therefore, in vitro-derived collateral sensitivity networks may not fully recapitulate evolutionary trajectories during infection. Future studies incorporating dynamic treatment designs in animal models will be required to evaluate the translational potential of collateral sensitivity-based strategies. Moreover, the present study was performed using experimentally evolved *E. coli* populations, and validation in a broader collection of clinical carbapenem-resistant *E. coli* (CRE) isolates remains necessary to determine the prevalence and clinical relevance of the identified collateral sensitivity mechanisms. Clinical isolates often exhibit diverse resistance determinants and compensatory adaptations that may modulate membrane homeostasis, efflux activity, and antibiotic susceptibility phenotypes. Future studies integrating genetically diverse clinical CRE isolates will be important to assess whether the mechanisms identified here can be generalized and exploited for therapeutic resensitization strategies.

## 5. Conclusions

In summary, this study identifies a robust collateral sensitivity to amphenicols that accompanies MEM resistance evolution in *E. coli*. We demonstrate that *yghB* disruption compromises membrane integrity and PMF homeostasis, resulting in impaired efflux, altered Mg^2+^ homeostasis, and ribosome-associated changes that contribute to increased intracellular accumulation of amphenicols. These interconnected physiological changes collectively drive hypersensitivity to amphenicols and create a collateral sensitivity-associated vulnerability with potential therapeutic relevance in MEM-resistant populations. Our findings advance the mechanistic understanding of collateral sensitivity and may inform the future development of evolution-informed strategies against multidrug-resistant Gram-negative pathogens.

## Figures and Tables

**Table 1:** Antibiotics used in this study.

Antibiotic	Abbreviation	Class	Target
Cefotaxime	CTX	Cephalosporin	Cell wall
Ceftiofur	CEF	Cephalosporin	Cell wall
Meropenem	MEM	Carbapenem	Cell wall
Levofloxacin	LVF	Quinolone	DNA gyrase
Ciprofloxacin	CIP	Quinolone	DNA gyrase
Apramycin	APR	Aminoglycoside	Protein synthesis, 30S
Amikacin	AMK	Aminoglycoside	Protein synthesis, 30S
Doxycycline	DOX	Tetracycline	Protein synthesis, 30S
Tetracycline	TET	Tetracycline	Protein synthesis, 30S
Chloramphenicol	CHL	Amphenicol	Protein synthesis, 50S
Florfenicol	FFC	Amphenicol	Protein synthesis, 50S
Sulfamethoxazole/Trimethoprim	SMX/TMP	Sulfonamide	Folic acid synthesis
Fosfomycin	FOS	Fosfomycin	Cell wall biogenesis
Colistin	COL	Polymyxin	Lipopolysaccharide

**Table 2:** Minimum inhibitory concentrations of deletion mutants Δ*envZ*, Δ*ynfL*, Δ*yghB*, Δ*envZ*Δ*yghB* and Δ*ynfL*Δ*yghB*.

MIC (mg/L)	WT	Δ*envZ*	Δ*ynfL*	Δ*yghB*	Δ*envZ*Δ*yghB*	Δ*ynfL*Δ*yghB*
MEM	0.06	0.06	0.06	0.06	0.06	0.06
CHL	256	256	128	32	64	64
FFC	4	4	2	0.5	1	1
SMX/TMP	10	5	5	5	1.2	5
CTX	256	256	256	256	128	128
CEF	256	256	256	256	128	128
DOX	0.5	0.5	0.5	1	2	0.5
TET	1	1	1	2	8	1
APR	4	4	4	4	1	1
AMK	2	2	2	2	0.5	0.5
COL	0.5	0.5	0.5	0.5	1	1

## Data Availability

The original data presented in the study are openly available in the National Center for Biotechnology Information (NCBI) at https://www.ncbi.nlm.nih.gov/, accession number PRJNA1224518, PRJNA1224587 and PRJNA1226379.

## Supplementary Materials

The following supporting information can be downloaded at: https://www.mdpi.com/article/10.3390/microorganisms14081792/s1, Figure S1. Growth characteristics of WT, Δ*yghB*, Δ*yghB*::*yghB*^WT^, and Δ*yghB*::*yghB*^FS^ strains under non-selective conditions. Relative maximum growth rate (A), area under the growth curve (AUC) (B) and lag phase duration (lag time) (C) of different *yghB*-associated strains; Figure S2. Transcriptomic profiling reveals physiological remodeling during MEM adaptation. (A) KEGG enrichment analysis of differentially expressed genes in MEM-R relative to the WT. (B) Differentially expressed genes associated with transporter families, PTS and cytochrome oxidase in MEM-R; Figure S3. Differentially expressed genes associated with ribosomal synthesis, membrane homeostasis and transporter proteins in Δ*yghB* mutants; Figure S4. Effect of Mg^2+^ supplementation (50 mM) on membrane potential (A) and membrane function (B-C) in WT and Δ*yghB* mutants; Figure S5. Effects of bicarbonate supplementation on the transmembrane proton gradient (ΔpH, A) and membrane potential (B); Figure S6. The *yghB* mutation reduces cardiolipin content; Table S1. Bacterial strains used in this study; Table S2. Plasmids used in this study; Table S3. Oligonucleotides primers used in this study; Table S4. Minimum inhibitory concentrations of parental strain and evolved populations; Table S5. Mutations identified in MEM-resistant *E. coli* strains isolated from the experimental evolution study.

## Abbreviations

The following abbreviations are used in this manuscript:

*E. coli*	*Escherichia coli*
WT	*E. coli* strain C600 with an IncI1 clinical plasmid p15E179 carrying *catA1* and *bla*_CTX-M-55_
MEM-R	Meropenem-selected evolved populations derived from the WT strain
MEM	Meropenem
CHL	Chloramphenicol
FFC	Florfenicol
CIP	Ciprofloxacin
FOS	Fosfomycin
DOX	Doxycycline
APR	Apramycin
COL	Colistin
PMF	Proton motive force
MIC	Minimum inhibitory concentration
