# Supplementary material for: Impaired Membrane Energetics Underlies Collateral Sensitivity to Amphenicols During Meropenem Resistance Evolution in Escherichia coli
Source: Microorganisms. 2026 Aug 14;14(8):1792. doi: 10.3390/microorganisms14081792 (PMC13515844; doi:10.3390/microorganisms14081792)
Supplement: Supplementary file 1 [file microorganisms-14-01792-s001.zip › Fig S1-S6.pdf]

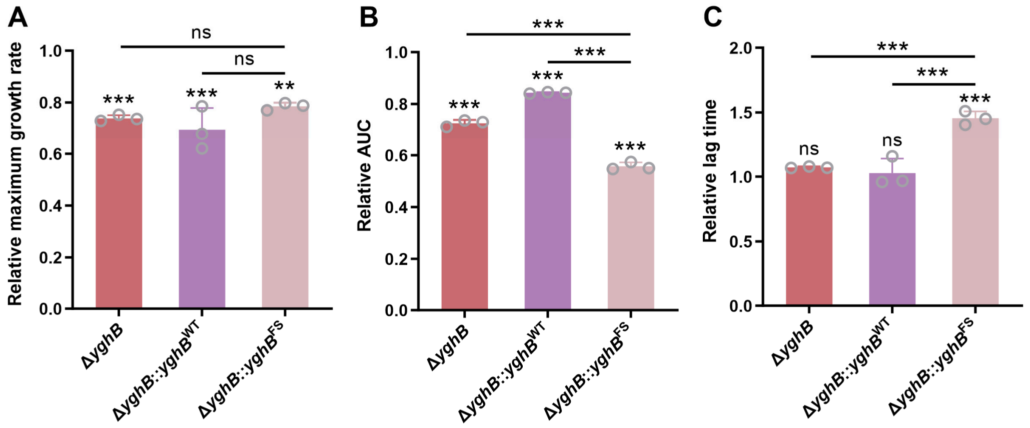

Figure S1. Growth characteristics of WT, Δ*yghB*, Δ*yghB*::*yghB*^{WT}, and Δ*yghB*::*yghB*^{FS} strains under non-selective conditions. Relative maximum growth rate (A), area under the growth curve (AUC) (B) and lag phase duration (lag time) (C) of different *yghB*-associated strains.

Bacterial cultures were diluted to an initial OD_{600} of 0.05 in fresh LB medium and incubated at 37°C with continuous shaking. Growth was monitored by measuring OD_{600} at regular intervals for 24 h. Growth parameters, including maximum growth rate, AUC and lag time were calculated from growth curves. The maximum growth rate and AUC were calculated using the Growthcurver package in R. The lag time was determined using Origin 2021 (OriginLab Corporation, Northampton, MA, USA).

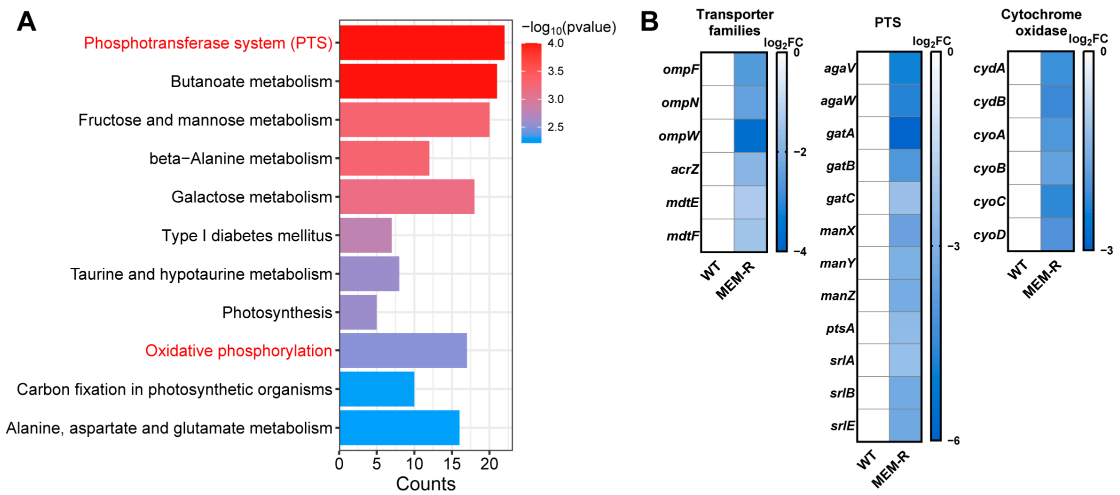

Figure S2. Transcriptomic profiling reveals physiological remodeling during MEM adaptation. (A) KEGG enrichment analysis of differentially expressed genes in MEM-R relative to the WT. (B) Differentially expressed genes associated with transporter families, PTS and cytochrome oxidase in MEM-R.

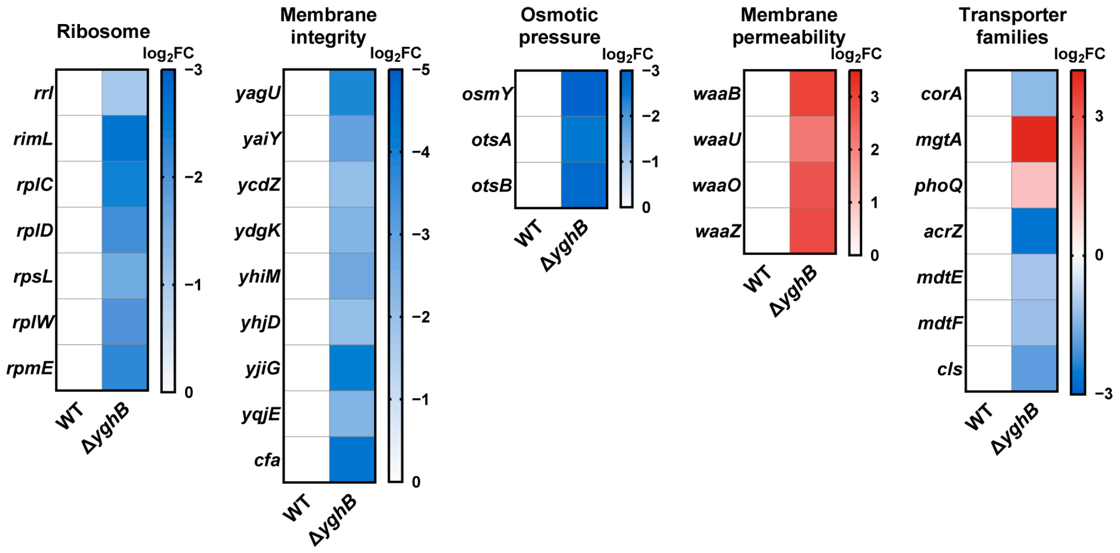

Figure S3. Differentially expressed genes associated with ribosomal synthesis, membrane homeostasis and transporter proteins in Δ*yghB* mutants.

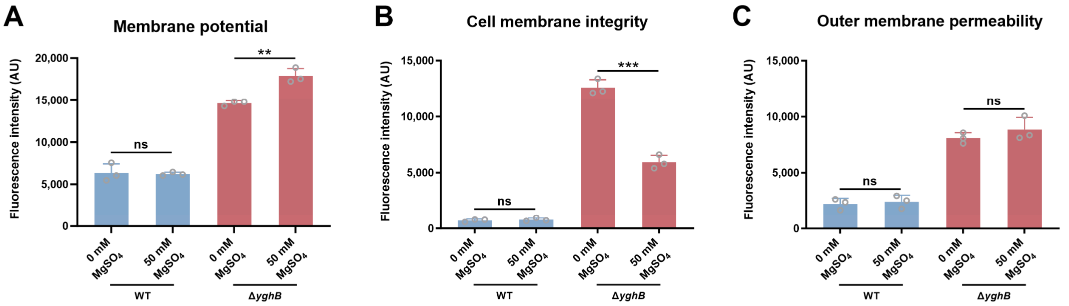

Figure S4. Effect of Mg^{2+} supplementation (50 mM) on membrane potential (A) and membrane function (B-C) in WT and Δ*yghB* mutants.

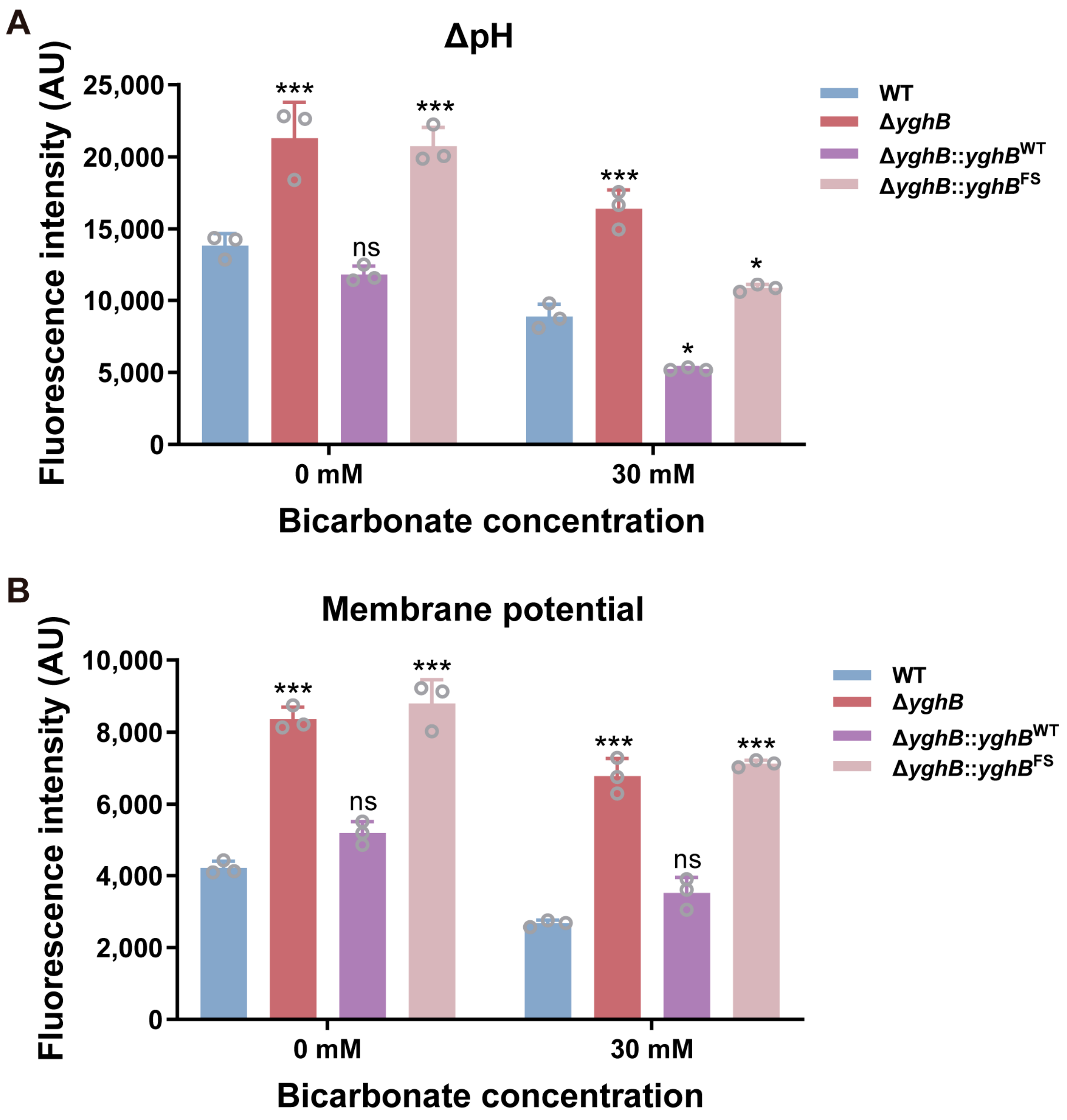

Figure S5. Effects of bicarbonate supplementation on the transmembrane proton gradient (ΔpH, A) and membrane potential (B).

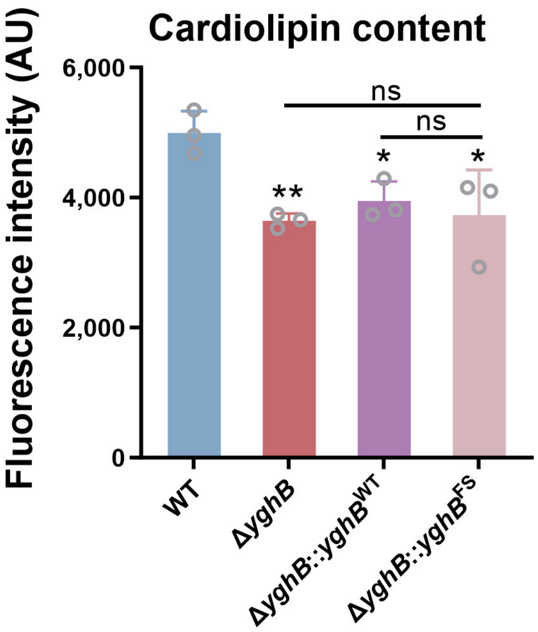

Figure S6. The *yghB* mutation reduces cardiolipin content.

Bacterial suspensions were stained with nonyl acridine orange (NAO) and incubated at 37°C for 1 h. Subsequently, 200 μL of the bacterial solution was transferred to a 96-well plate and measured in the dark. Fluorescence intensity (excitation = 498 nm, emission = 521 nm) was used to assess the transmembrane proton gradient and membrane potential.
