# Supplementary material for: Impaired Membrane Energetics Underlies Collateral Sensitivity to Amphenicols During Meropenem Resistance Evolution in Escherichia coli
Source: Microorganisms. 2026 Aug 14;14(8):1792. doi: 10.3390/microorganisms14081792 (PMC13515844; doi:10.3390/microorganisms14081792)
Supplement: Supplementary file 1 [file microorganisms-14-01792-s001.zip › TableS.pdf]

Table S1. Bacterial strains used in this study.

Strains	Description	Sources
C600	*E. coli* strain.	Lab collection
WT	*E. coli* strain C600 with an IncI1 clinical plasmid p15E179 carrying *catA1* and $bla_{CTX-M-55}$.	This study
MEM-R	Meropenem-selected evolved populations derived from the WT strain.	This study
WT::pBAD	WT with pBAD.	This study
WT::$mrdA^{WT}$	WT with pBAD::$mrdA^{WT}$.	This study
WT::$mrdA^{MEM-R}$	WT with pBAD::$mrdA^{MEM-R}$.	This study
WT::$envZ^{WT}$	WT with pBAD::$envZ^{WT}$.	This study
WT::$envZ^{MEM-R}$	WT with pBAD::$envZ^{MEM-R}$.	This study
WT::$envZ^{MEM-R}mrdA^{MEM-R}$	WT with pBAD::$envZ^{MEM-R}mrdA^{MEM-R}$.	This study
Δ*envZ*	WT with deletion of *envZ*.	This study
Δ*ynfL*	WT with deletion of *ynfL*.	This study
Δ*yghB*	WT with deletion of *yghB*.	This study
Δ*envZ*Δ*yghB*	WT with deletion of *envZ* and *yghB*.	This study
Δ*ynfL*Δ*yghB*	WT with deletion of *ynfL* and *yghB*.	This study
Δ*yghB*::$yghB^{WT}$	Δ*yghB* with pBAD::$yghB^{WT}$.	This study
Δ*yghB*::$yghB^{FS}$	Δ*yghB* with pBAD::$yghB^{FS}$.	This study
WT^{FS}	Chromosomal reconstruction of the evolved *yghB* frameshift allele (A645del)	This study

Table S2. Plasmids used in this study.

Plasmids	Genotype or phenotype	Sources
pBAD24	oriVp15A; Para (KanR)NcoI site of expression vector pBAD24 changed to an NdeI site.	Lab collection
pBAD::*mrdA*^{WT}	pBAD24 carrying *mrdA* from WT.	This study
pBAD::*mrdA*$^{MEM-R}$	pBAD24 carrying *mrdA* from MEM-R.	This study
pBAD::*envZ*^{WT}	pBAD24 carrying *envZ* from WT.	This study
pBAD::*envZ*$^{MEM-R}$	pBAD24 carrying *envZ* from MEM-R.	This study
pBAD::*envZ*$^{MEM-R}$*mrdA*$^{MEM-R}$	pBAD24 carrying *envZ* and *mrdA* from MEM-R.	This study
pCasKP-apr	Thermosensitive plasmid, expresses Cas9 and lambda proteins in *E. coli* (AprR)	Lab collection
pSGKP-rif	Plasmid expressing sgRNA in *E. coli* (RifR)	Lab collection
pSGKP_*envZ*	Plasmid expressing sgRNA of *envZ* in *E. coli* (RifR)	This study
pSGKP_*envZ*_UD	pSGKP-rif derivative with *envZ* spacer and ~0.5 kb each repair arm	This study
pSGKP_*ynfL*	Plasmid expressing sgRNA of *ynfL* in *E. coli* (RifR)	This study
pSGKP_*ynfL*_UD	pSGKP-rif derivative with *ynfL* spacer and ~0.5 kb each repair arm	This study
pSGKP_*yghB*	Plasmid expressing sgRNA of *yghB* in *E. coli* (RifR)	This study
pSGKP_*yghB*_UD	pSGKP-rif derivative with *yghB* spacer and ~0.5 kb each repair arm	This study
pSGKP_*yghB*_UD2	pSGKP-rif derivative with *yghB* spacer and kanamycin cassette with ~0.5 kb each repair arm	This study
pSGKP_FS	Plasmid expressing sgRNA of kanamycin in *E. coli* (RifR)	This study
pSGKP_FS_UD	pSGKP-rif derivative with *yghB* spacer and the *yghB* frameshift allele with ~0.5 kb each repair arm	This study
pBAD::*yghB*^{WT}	pBAD24 carrying *yghB* from WT.	This study
pBAD::*yghB*^{FS}	pBAD24 carrying *yghB* from MEM-R.	This study
pET28a (+)	Expression vector pET28a (+) (KanR).	Lab collection
pET28a_*yghB*^{WT}	pET28a (+) carrying *yghB* from WT.	This study
pET28a_*yghB*^{FS}	pET28a (+) carrying *yghB* from MEM-R.	This study

Table S3. Oligonucleotides primers used in this study.

**Primers**	**Sequences (5'-3')**
pBAD-FF	gaattcctcctgctagccc
pBAD-FR	acccggggatcctctaga
pBAD_*mrdA*-F	gggctagcaggaggaattcatgaaactacagaactcttttcgcg
pBAD_*mrdA*-R	tctagaggatccccgggtttaatggtcctccgctg
pBAD_*envZ*-F	gggctagcaggaggaattcatgaggcgattgcgcttctcgc
pBAD_*envZ*-R	tctagaggatccccgggtttacccttcttttgtcgtgcc
pBAD_*envZmrdA*-F	acgacaaaagaagggtaaatgaaactacagaactct
pBAD_*envZmrdA*-R	gagttctgtagtttcatttacccttcttttgtcgtgcc
pBAD-test-F	cacggcgtcacactttgc
pBAD-test-R	cagaccgcttctgcgttc
pSGKP_*envZ*-HF	agtgaacacgcagccttgcaggtgttttagagctagaaatagcaag
pSGKP_*envZ*-HR	aaacacctgcaaggctgcgtgttcactagtattatacctaggactgag
pSGKP_*envZ*-UF	tgatatcgaattcctgcagcccgggatgcgcctgcgtgcgctgct
pSGKP_*envZ*-UR	ggaggcaccttcgcctcccgtttatgctttagagccgtccggtac
pSGKP_*envZ*-DF	ataaacgggaggcgaaggtgcctcc
pSGKP_*envZ*-DR	tagaagtagtggatcccccgggtcgataacattgttaagccggtt
envZ-test-F	accaggggcgttttcatctcgt
envZ-test-R	acaacgctatccgtcgtgatgc
pSGKP_*ynfL*-HF	agtgcgaaacagcgataacgtatgttttagagctagaaatagcaag
pSGKP_*ynfL*-HR	aaacatacgttatcgctgtttcgcactagtattatacctaggactgag
pSGKP_*ynfL*-UF	tgatatcgaattcctgcagcccggggtacaaatggaggccaacag
pSGKP_*ynfL*-UR	tcgggctttttcgctgaaatttcccatttaaaacatcttatttga
pSGKP_*ynfL*-DF	gggaaatttcagcgaaaaagcccga
pSGKP_*ynfL*-DR	tagaagtagtggatcccccggggctcagatcgcgcagagcaa
ynfL-test-F	actgggatggatttcctcgcta
ynfL-test-R	caatcgaagttagacgctcaag
pSGKP_*yghB*-HF	agtactggttaagtggattgctggttttagagctagaaatagcaag
pSGKP_*yghB*-HR	aaaccagcaatccacttaaccagtactagtattatacctaggactgag
pSGKP_*yghB*-UF	tgatatcgaattcctgcagcccgggtattcagcatggcctactcg
pSGKP_*yghB*-UR	cagcaaccgggaacggggaaaatcgggactttcctgtggaacagc
pSGKP_*yghB*-DF	cgattttccccgttcccggttgctg
pSGKP_*yghB*-DR	tagaagtagtggatcccccgggtacaccgaaaacctgagcgtcga
pSGKP_*yghB*-UR2	cgtgcaatccatcttgttcaatcatggactttcctgtggaacagc
Kan-F	ccacaggaaagtccatgattgaacaagatggattgc
Kan-R	ccgggaacggggaaaatcgtcagaagaactcgtcaa

pSGKP_*yghB*-DF2	gacgagttcttctgacgattttccccgttcccggttgctg
pSGKP-FS-HF	agtagaggctattcggctatgacgttttagagctagaaatagcaag
pSGKP-FS-HR	aaacgtcatagccgaatagcctctactagtattatacctaggactgag
pSGKP_FS-UR	tcttgaataacagccatggactttcctgtggaacagc
FS-F	acaggaaagtccatggctgttattcaagatatcatc
FS-R	ggggaaaatcgtcaggcgttacagtatttttttta
pSGKP_FS-DF	tgtaacgcctgacgattttccccgttcccggttgctg
yghB-test-F	cctggcagtaaaggtcacgaat
yghB-test-R	tttgagtgcgaaacctatgcca
pSGKP-test-F	tctcgtttggattgcaactg
pSGKP-test-R	tgcttccggctcgtatgttg
pBAD_*yghB*-F	gggctagcaggaggaattccattgccacttttggacaattttgc
pBAD_*yghB*-R	tctagaggatccccgggtatacgttcccggtacagcaacc
pBAD_*yghB*^{FS}-R	tctagaggatccccgggtttaaatacgttcccggtacagcaacc
pet-*yghb*-F	catgactggtggacagcaaatgggtatggctgttattcaagatatcatcgctgcgc
pet-*yghb*-R	tcgacggagctcgaattcggatccgcgtcaggcgttacagtattttttttaatca
pet-*yghb*^{FS}-R	tcgacggagctcgaattcggatccgcgttaaatacgttcccggtacagcaaccgggaa
pet28a-FF	acccatttgctgtccacc
pet28a-FR	cgcggatccgaattcgag
pet28a-test-F	cgtccggcgtagaggatcgagatc
pet28a-test-R	atccggatatagttcctcctttc
qp-16S-F	attagataccctggtagtccacgc
qp-16S-R	ttgcgggacttaacccaac
qp-*corA*-F	tctgtatcgtatgcgtgcccgt
qp-*corA*-R	cgctgggtatccatcagacaca
qp-*mgtA*-F	gaagagggggttattgagggac
qp-*mgtA*-R	cgccacctgtgacacatcgtac
qp-*phoQ*-F	acccttgcgaagtgggaaaaca
qp-*phoQ*-R	gctgcccgttctcatcataaat
qp-*rrl*-F	tgtgtgttagtggaagcgtctg
qp-*rrl*-R	tatttagccttggaggatggtc
qp-*rimL*-F	aggcatcattcaggaactcagc
qp-*rimL*-R	tactggctggacgaatctcatc
qp-*rplC*-F	agacctggctaacgatggctac
qp-*rplC*-R	cctacagtgaactcttcgcctt
qp-*rplD*-F	tctggttctatcaagagcccga
qp-*rplD*-R	accagttcggacaggatgcttt
qp-*rpsL*-F	cactcctacaaaaccgaactcc

qp-*rpsL*-R	ttcacgccatacttggaacgag
qp-*rplW*-F	tctgaaaaagcgtctactgcga
qp-*rplW*-R	tccgtgacgtttaactttccct
qp-*rpmE*-F	cccgggatgttgaaacgcttgt
qp-*rpmE*-R	atgaaaatccgctccaccgttg

Note: FF: Reverse amplification primers.

Table S4-1. Minimum inhibitory concentrations of parental strain and evolved populations.

MIC	Parental		MEM						CIP						FOS					
(mg/L)	WT	C600	WT			C600			WT			C600			WT			C600		
CTX	256	0.03	1	2	1	0.1	1	0.1	512	512	512	0.5	0.1	0.1	512	64	256	0.5	0.1	0.1
CEF	256	0.06	2	2	2	0.5	0.1	0.06	128	128	128	0.2	0.1	0.06	256	32	128	0.5	0.2	0.5
MEM	0.06	0.06	8	8	8	8	16	4	0.06	0.06	0.06	1	0.2	0.5	0.06	0.06	0.06	0.5	0.5	0.5
LVF	0.03	0.03	1	2	1	0.007	0.06	0.0009	0.5	1	0.5	2	2	2	0.03	0.03	0.03	0.007	0.0009	0.03
CIP	0.03	0.03	1	2	1	0.06	0.03	0.06	2	4	2	4	512	256	0.03	0.03	0.03	0.06	0.06	0.06
APR	4	4	2	2	2	4	4	4	8	8	4	4	4	4	2	4	2	4	4	4
AMK	2	2	2	2	2	2	2	4	2	2	2	2	4	4	1	1	1	2	4	2
CHL	256	4	4	4	4	1	2	2	512	512	512	8	8	8	512	512	512	4	4	4
FFC	4	4	0.06	0.06	0.06	1	2	2	8	8	8	8	8	8	8	8	8	4	4	4
SMX/TMP	10	10	0.6	0.6	0.6	2.5	10	10	2.5	5	10	40	20	10	2.5	5	5	5	2.5	2.5
FOS	1	1	0.5	0.5	1	0.2	4	4	0.5	0.5	0.5	4	32	4	256	256	256	64	64	64
COL	0.5	0.5	1	1	1	1	2	0.5	0.5	0.5	0.5	128	128	1	0.5	0.5	0.5	1	1	1
DOX	0.5	0.5	1	0.5	0.5	8	8	8	4	4	8	32	8	16	2	8	4	4	4	4
TET	1	1	1	1	1	2	4	2	4	4	8	16	8	8	16	16	16	32	2	32

Table S4-2. Minimum inhibitory concentrations of parental strain and evolved populations.

**MIC**	**Parental**		**DOX**						**APR**						**COL**					
**(mg/L)**	**WT**	**C600**	**WT**			**C600**			**WT**			**C600**			**WT**			**C600**		
CTX	256	0.03	128	128	256	1	1	0.5	256	128	128	0.06	0.06	0.06	256	256	64	0.1	0.1	0.1
CEF	256	0.06	16	32	64	0.5	0.5	0.5	64	32	64	0.007	0.06	0.06	128	256	2	0.5	0.1	0.06
MEM	0.06	0.06	0.06	0.06	0.06	0.5	0.5	0.5	0.06	0.06	0.06	0.5	0.25	0.25	0.06	0.06	0.06	0.5	0.5	0.5
LVF	0.03	0.03	0.03	0.03	0.03	0.01	2	2	0.03	0.03	0.03	0.0009	0.0009	0.0009	0.03	0.03	0.03	0.1	2	2
CIP	0.03	0.03	0.2	0.2	0.2	0.25	0.25	0.25	0.03	0.03	0.03	0.06	0.06	0.06	0.03	0.03	0.03	0.06	0.1	0.06
APR	4	4	4	8	8	2	2	2	256	64	128	64	8	32	8	32	2	8	8	8
AMK	2	2	0.5	0.5	0.5	1	4	2	32	16	32	64	32	16	2	32	1	4	4	4
CHL	256	4	256	256	256	8	8	8	16	16	16	0.5	0.5	0.5	16	64	32	1	0.5	0.5
FFC	4	4	4	4	4	8	8	8	0.2	0.2	0.2	0.5	0.5	0.5	0.2	1	0.5	1	0.5	0.5
SMX/TMP	10	10	5	2.5	640	20	20	40	2.5	2.5	2.5	5	10	10	2.5	2.5	2.5	5	5	2.5
FOS	1	1	1	1	1	8	1	4	2	2	2	4	4	1	1	1	4	4	4	8
COL	0.5	0.5	0.2	2	0.2	0.5	1	1	0.2	0.2	0.2	16	8	32	64	32	32	128	512	256
DOX	0.5	0.5	32	32	16	256	256	256	2	2	1	2	2	2	1	1	1	2	4	2
TET	1	1	16	16	16	64	64	64	8	8	8	2	2	2	0.5	0.5	0.5	2	4	2

Table S5. Mutations identified in MEM-resistant *E. coli* strains isolated from the experimental evolution study.

**MEM-resistant *E. coli***	**Gene**	**Nucleotide change**	**Effect of nucleotide change**
WT	*yghB*	A645Del	K215fs
	ynfL	G17T	R6L
	envZ	C700T	R234C
	mrdA	G1802A	G601D
C600	*yghB*	A645Del	K215FS
	envZ	T104G	L35P
	mrdA	G1513A<br>A1639G	G505S<br>T547A
	btuB	A1024G	T342A
	mdtM	T1133del	L378R

Del: deletion. FS: frameshift.
